# Impact of Teachers’ Post-Traumatic Stress Due to Violence Victimization: Moderated Mediation Effect of Living a Calling

**DOI:** 10.3390/bs13020139

**Published:** 2023-02-07

**Authors:** Yiran Li, Jeehyon Ahn, Sein Ko, Inchi Hwang, Youngseok Seo

**Affiliations:** 1 Institute for Educational Research, Faculty of Education, Yonsei University, 50, Yonsei-ro, Seodaemun-gu, Seoul 03722, Republic of Korea; 2 Faculty of Education, Yonsei University, 50, Yonsei-ro, Seodaemun-gu, Seoul 03722, Republic of Korea

**Keywords:** violence against teachers, post-traumatic stress disorder, social interaction anxiety, psychological burnout, self-destructive behaviors, turnover intention

## Abstract

Based on the Affective Events Theory, Work as a Calling Theory, and related studies, this research examined the moderated mediating effects of Living a Calling and the mediating effect of social interaction anxiety and psychological burnout on the relationships between post-traumatic stress disorder symptoms consequent to violence victimization, self-destructive behaviors, and turnover intention. Data from 420 Korean elementary and secondary school teachers were analyzed using the moderated mediation model. The analysis revealed that post-traumatic stress disorder caused by violence victimization positively affected self-destructive behavior and turnover intention through the sequential mediation of social interaction anxiety and psychological burnout. Further, Living a Calling moderated the indirect effect of violence victimization; the stronger the Living-a-Calling experience, the greater the indirect effect of violence victimization on turnover intention. Additionally, when the sense of Living a Calling was low, post-traumatic stress disorder caused by violence did not significantly affect turnover intention through social interaction anxiety, but contrary to expectations, the stronger the sense of Living a Calling, the more positive the mediating effect of social interaction anxiety. Therapeutic interventions in teachers’ work environment, improvements, and suggestions for future research are discussed.

## 1. Introduction

Teacher victimization in student violence is a serious issue that has a grave impact on teachers’ decisions to transfer to another school or leave the profession altogether [1]. According to Korean education statistics, 10,531 teachers quit the profession in 2020; this included retirement (n = 3198; 30.4%), voluntary early retirement (n = 6164; 58.5%), and other reasons and voluntary resignation (n = 1169; 11.1%), where teachers leaving their jobs before retirement age (e.g., voluntary early retirement) far outnumbered those who retired at retirement age [2]. Further, according to the Korean Federation of Teachers’ Association [3], 402 cases of counseling pertaining to the infringement of teachers’ rights were submitted and processed in 2020, showing that a substantial number of teachers suffered mental distress as a result of such infringement. Student violence against teachers not only threatens teachers’ mental health but also takes a toll on teachers’ overall lives, including their physical health and interpersonal relationships [4]. Student violence research thus far has primarily been focused on student victims. Although some studies have begun to focus on teachers in recent years, such studies are still at a rudimentary stage [5]. Identifying the psychological mechanisms underlying the victimization of teachers by students is important for exploring measures to develop a policy regarding therapeutic interventions to help the teachers who have been victimized.

Teachers who fall victim to student violence experience post-traumatic stress disorder (PTSD) [6]. These teachers may continuously relive the trauma, avoid interactions with others, and react over-sensitively to students’ problem behaviors [7]. Particularly, teacher victims of violence expressed mistrust and fear of students and parents and stated that their relationships with their colleagues, including the principal, deteriorated [4]. Furthermore, infringement of teachers’ rights, including student violence against teachers, physically and psychologically, exhausts teachers, leads to them maintaining a cold and indifferent attitude toward students, and curtails their sense of accomplishment as a teacher, resulting in psychological burnout [8]. Moreover, PTSD symptoms triggered by violence may lead to self-destructive behavior, such as binge-eating and alcohol-use problems [9,10,11,12]. These PTSD symptoms also undermine teachers’ confidence in their jobs [13], causing them to start doubting the teaching profession and consider leaving their jobs [7]. In essence, elementary, middle, and high school teachers experience PTSD symptoms after falling victim to student violence. This has been speculated to influence relationship anxiety, psychological burnout, self-destructive behaviors, and turnover intention. In the present study, we investigated whether PTSD symptoms triggered by exposure to violence affect teachers’ self-destructive behaviors and, ultimately, turnover intention, resulting from social interaction anxiety and psychological burnout. To develop a better understanding of these psychological mechanisms, self-destructive behaviors, and turnover intention among teachers, we established a structural model on teacher victimization with violence—the factor previously reported to be the most potent predictor of teachers’ resignations and self-destructive behaviors—together with PTSD, social interaction anxiety, and psychological burnout based on the affective events theory (AET), and we evaluated the potential moderating effects of a psychological variable called “Living a Calling”.

In this study, the AET has been a useful theory explaining the causal relationships between human cognition, emotion, attitude, and behavior. It has been utilized in various human conditions, including school violence, workplace bullying, trauma, psychodynamics, anxiety disorders, counseling, and psychological therapy [14,15]. According to this theory, being exposed to an adverse event (e.g., violence) triggers PTSD as a cognitive emotional response to the event, which ultimately results in psychological problems. Teachers’ experience of student violence affects psychological problems pertaining to emotion and attitude (e.g., social interaction anxiety and psychological burnout), and the frustration and emotional distress caused by infringed teachers’ rights and the teaching profession per se can influence their decision to resign and their self-destructive behaviors. The theory posits that a positive or negative perception of an event (e.g., experiencing student violence that infringes teachers’ rights) can engender certain emotional and psychological problems (e.g., PTSD) or attitudes (e.g., social interaction anxiety and psychological burnout), and these, in turn, lead to certain behaviors (e.g., turnover intention and self-destructive behavior).

Based on the AET and existing literature, Karatuna and Gok [15] and Weiss and Cropanzano [16] proposed a “cognition → emotion → attitude → behavior” model that explains how teachers’ emotional response to a situation encountered in school or a job-related event fosters and determines their attitude and behavior. Hence, if teachers experience psychological problems in their personal and professional lives as a result of PTSD caused by their victimization from school violence [9], they may experience emotional changes, such as anger, embarrassment, and communication problems, and develop social interaction anxiety, sleep disorder, depression, emotional distress, and psychological burnout, ultimately increasing their tendency to resign and their self-destructive behavior [5,12]. Contractor and Weiss [17] confirmed that PTSD from having suffered violence affects turnover intention and self-destructive behavior, precedes social interaction anxiety, and predicts certain psychological mechanisms. Therefore, social interaction anxiety increases with increasing PTSD symptoms, intensifying psychological burnout, thereby promoting turnover intention and self-destructive behaviors [18]. In light of the AET, this study aimed to understand whether victimization through violence increases the PTSD symptoms and the consequent psychological mechanisms, self-destructive behavior, and turnover intention among education teachers who have had their rights as a teacher infringed, particularly through student violence.

### 1.1. PTSD Caused by Teacher Victimization with Student Violence: Beginning of Self-Destructive Behaviors and Turnover Intention

Previous studies [4,19,20] have defined PTSD suffered by teachers who experienced student violence as difficulties experienced while imparting their educational philosophy and values to students and their consequent demonstration of negative cognitive, emotional, attitude, and behavioral responses accompanied by chronic anxiety and depression. Teachers who frequently experienced violence in school feared the possibility of another violent incident [4,19]. They developed symptoms of PTSD, where they repeatedly engaged in a harmful re-living of the adverse event and were left without ways to overcome the trauma; all events were perceived as threatening and the cycle was perpetuated unless the stress was relieved [21].

The related literature emphasizes that PTSD is responsible for psychological problems and self-destructive behaviors, including turnover intention among basic education teachers. Specifically, prior studies examined the association between student violence and teachers’ psychological burnout [8], shed light on increasing alcohol use with increasing PTSD among workers [22], and investigated the relationship between PTSD and turnover intention in firefighters [23].

However, there is a dearth of studies examining the relationships between PTSD caused by student violence against teachers and social interaction anxiety, psychological burnout, and its association with self-destructive behavior and turnover intention. Studies conducted on elementary, middle, and high school teachers examining the predictors of PTSD and the causes of burnout and traumatic experience have been limited, with not many studies exploring the effects of PTSD from a violent incident [24,25,26]. Specifically, studies have rarely examined the relationship of PTSD caused by student violence against teachers with PTSD per se and self-destructive behaviors. Referring to previous study findings pertaining to PTSD, which is closely linked to self-destructive behavior [5,9,27,28], we hypothesized that PTSD influences self-destructive behavior. In terms of turnover intention, some studies reported that teachers who have PTSD experience psychological burnout and develop the intention to leave their jobs [29,30], but not many studies have investigated the relationship of PTSD from a violent incident with psychological problems and self-destructive behavior with potentially adverse outcomes, as well as the turnover intention with PTSD as the predictor. In essence, PTSD may increase self-destructive behavior and turnover intention.

### 1.2. Social Interaction Anxiety and Psychological Burnout: CATALYSTS of Self-Destructive Behavior and Turnover Intention

PTSD from student violence can be associated with teachers’ self-destructive behavior and turnover intention for the following reasons. First, based on research findings [24,31,32,33,34,35], PTSD may bring about psychological problems in teachers through social interaction anxiety, which can increase their self-destructive behaviors and intention to resign. Numerous situational factors or triggers in school may cause job stress, psychological problems, and anxiety among teachers [32], and in such situations, teachers can experience social interaction anxiety. Social interaction anxiety was first introduced by Mattick and Clarke [36]. It was defined as anxiety or fear of making a fool out of oneself, being ignored, or being considered boring when meeting and interacting with other people [33]. If teachers develop PTSD due to exposure to violence, they experience anxiety in social interactions, which engenders psychological problems such as depression and fear [17,22,37]. Further, they fear engaging in interpersonal relationships and potentially encounter difficulties in relationships [38].

Past studies on the infringement of teachers’ rights [4,5,39] reported that frequent verbal abuse in the workplace, compensation incommensurate to the position, and poor work environment not only reduce teaching self-efficacy but also increase teachers’ post-traumatic stress, thereby increasing the risk of anxiety during social relationships and interactions. Parallelly, several qualitative studies [29,30] reported that the infringement of teachers’ rights increased their doubts about their profession and their frustration, and was, consequently, linked to their anxiety in teacher–student relationships and other interpersonal relationships. Based on these findings, the severity of social interaction anxiety is believed to increase with the increasing severity of PTSD among teachers.

Social interaction anxiety influences self-destructive behaviors such as binge-eating and alcohol use among teachers, and this can ultimately motivate teachers to leave their job. Social interaction anxiety disrupts direct communication and actions to solve problems [40], and escalates the level of worry and anxiety [24,34,40,41,42]. In particular, social interaction anxiety can intensify teachers’ worry about various situations, including their job, professional efficacy, goals, and future plans, besides significantly impacting their personal lives [30,43]. Student violence against teachers—an act of infringement of teachers’ rights—overwhelms teachers with negative emotions, arouses anxiety in their social interactions, and may facilitate avoidance behaviors among them, such as quitting their job and seeking immediate gratification in ways that ultimately incur harm, such as binge-eating and alcohol use [28,35]. Thus, teachers who have fallen victim to violence may engage in binge-eating, use alcohol, and decide to leave their jobs due to the effects of social interaction anxiety [28,44].

Teachers with PTSD due to violence and infringed rights may also experience psychological burnout as they feel psychologically overwhelmed in their jobs. Psychological burnout among teachers is defined as a negative cognitive and emotional response and attitude triggered by an event that induces job stress [8]. Thus far, studies investigating teachers’ psychological burnout [24,37,45,46,47,48] have used the fear of the infringement of teachers’ rights, victimization to violence, and social interaction anxiety as predictor variables. In this present study, we included psychological burnout in the model to examine whether PTSD from student violence and the consequent social interaction anxiety influence psychological burnout among basic education teachers.

In contrast to other stress events, a traumatic event can bring about psychological burnout in that it induces emotional shock, which victims have difficulty overcoming [49]. Thus, teachers who experience PTSD may also undergo psychological burnout during the process of overcoming their trauma [50,51,52,53]. A study that examined the causes of psychological burnout among teachers [54] reported that those with high stress in school or low emotional regulation exhibited the symptoms of psychological burnout. Another study [55] found that teachers who experienced PTSD demonstrated higher psychological burnout and depression during their interaction with students, as well as lower professional passion, confidence, and self-efficacy, which in turn led to self-destructive behaviors and turnover intention [28,56].

Lee [34] argued that job stress, emotional labor, and workload are the most potent causes of psychological burnout among teachers and that these factors eventually increase their turnover intention. Moreover, Lee and Lee [41] reported that teachers who feel that their authority as teachers had been violated or undermined began to doubt their respectability as teachers, as well as their efficacy and aptitude for the profession, leading to burnout and confusion about their ability to continue teaching. Elevating psychological burnout levels lowers teaching performance and resilience [57], and teachers with reduced professional efficacy may want to abandon the profession [34]. Therefore, we attempted to test the hypothesis that PTSD in teachers caused by exposure to student violence will exacerbate their psychological burnout, and thus, increase self-destructive behavior and turnover intention.

Social interaction anxiety may also increase teachers’ psychological burnout. Elevated social interaction anxiety causes teachers to doubt their aptitude for their profession and experience difficulties in teaching [24]. Teachers who experience severe social interaction anxiety are highly conscious of other people and perceive even normal anxiety in interpersonal relationships as threatening; they eventually experience negative emotions about themselves for not being able to handle the process well [24]. Furthermore, teachers who experience a high level of social interaction anxiety are excessively focused on regulating their psychological problems and negative emotions, which in turn may cause psychological burnout [58]. A qualitative study on resolving stress from the infringement of teachers’ rights [59] found that teachers who have social interaction anxiety and face challenges in interacting (e.g., talking and meeting) with other people (including students), develop an exaggerated perception of their inadequacies and experience psychological burnout. Thus, we aimed to test the hypothesis that basic education teachers with high social interaction anxiety may experience or perceive a greater level of psychological burnout in their teaching profession.

### 1.3. Living a Calling: Factors That Alleviate Self-Destructive Behavior and Turnover Intention

In recent years, Calling has been intensively studied to identify the predictors of job satisfaction and happiness among adult workers. The definition of a Calling varies across scholars, but one common aspect is that it is a source of a sense of fulfillment acquired from work itself, as work is in line with one’s purpose in life, as well as career identity that represents oneself [60,61,62]. According to some studies, people with a sense of Calling find meaning in their work and do not simply consider their work as a means to an end; they transcend the financial reward aspect and experience higher job satisfaction and relatively less job stress [63]. In a study involving adult workers, Calling was linked to low turnover intention [64,65,66] and high organizational commitment [44]. Thus, this study considered Calling as a protective factor against self-destructive behavior and turnover intention motivated by PTSD among teachers.

In the past, Calling was studied on the premise that it is present a priori, but in recent years, studies have begun to distinguish various aspects of Calling, such as Exploring a Calling, the Presence of a Calling, and Living a Calling [67,68,69], based on the notion that a Calling is not present all at once but is formed through a continuous process [68,69] and is subject to developmental variances. Out of the various constructs pertaining to Calling, Perceiving a Calling refers to being aware of one’s Calling and perceiving oneself to be Living the Calling [60,70,71]. In a study with teachers, Jang and Lee [70] showed that Perceiving a Calling alleviated PTSD or psychological problems in personal lives and school (which resulted from exposure to student violence), besides boosting life satisfaction. Conversely, teachers who did not perceive a Calling underwent a repetitive process in which they excessively relied on their psychological mechanisms to overcome emotional distress and depression amid social interaction anxiety and psychological burnout [39]. Those who perceived a Calling were found to have higher overall life satisfaction [71]. It can be inferred that teachers with a high sense of Calling strive to overcome emotional distress and depression even when students infringe on their rights as teachers. These teachers do not engage in self-destructive behaviors and develop turnover intention because their professional Calling and efficacy are strong [66,72,73,74].

A study that validated a structural model including Living a Calling and teacher burnout [75] also showed that Living a Calling moderated the process through which teachers’ burnout adversely affected job satisfaction, where high perceived Calling led to increased job satisfaction through increased professional efficacy and reduced psychological burnout. A study involving Korea Air Force cadets [76] found that grit has a greater impact on mental health among cadets with a higher perceived Calling; Choi [77] also showed that the effects of social interaction anxiety and psychological burnout on turnover intention were attenuated with an increased perception of Calling among teachers. In essence, Living a Calling affected turnover intention through interaction with social interaction anxiety and psychological burnout [34,76,77,78,79]. In this case, we can interpret that Living a Calling buffers or attenuates the psychological mechanism through which psychological burnout affects turnover intention. We can hypothesize that Living a Calling will attenuate the effects of social interaction anxiety and psychological burnout on self-destructive behaviors and turnover intention that result from diminished self-efficacy and the fear of negative evaluation among teachers [51].

Based on these findings, the effects of social interaction anxiety and psychological burnout on turnover intention are anticipated to be lower among teachers with high perceived Calling due to job satisfaction and self-efficacy; conversely, a stronger relationship may exist between social interaction anxiety and psychological burnout and turnover intention among teachers with low perceived Calling. People with high perceived Calling have a sense of duty to complete their tasks even if they feel burned out, resulting in declining job satisfaction as they carry out their tasks. Consequently, the adverse effects of psychological burnout on job satisfaction were found to be buffered or attenuated in these individuals [70,80]. In contrast, people with low perceived Calling are more likely to be emotionally exhausted or seek immediate gratification, such as through problem drinking and binge-eating, as well as leave their jobs due to psychological burnout experienced during work; hence, it can be inferred that the adverse effects of psychological burnout on job satisfaction will not be attenuated in these individuals. In sum, teachers may develop PTSD after exposure to student violence, which may heighten their social interaction anxiety and psychological burnout. However, teachers with high perceived Calling are more likely to be subject to the weaker effects of social interaction anxiety and psychological burnout on self-destructive behaviors and turnover intention, whereas teachers with low perceived Calling are more likely to be subject to the stronger effects of social interaction anxiety and psychological burnout on self-destructive behaviors and turnover intention.

Duffy et al. [81], who proposed the theory of Work as a Calling, argued that higher perceived Calling will strengthen the effects of work meaning and person–job fit on positive outcomes, such as job satisfaction and job performance, but may also strengthen the effects on negative outcomes, including work addiction and organizational exploitation. Therefore, Perceiving a Calling is clinically significant as a moderator that buffers the effects of social interaction anxiety and psychological burnout resulting from PTSD from violence victimization by students regarding teachers’ self-destructive behaviors and turnover intention, but excessively holding on to a perceived Calling may increase the risk of being exploited by the organization or developing work addiction, as hinted by the notion that an “inordinate sense of duty engenders a fatigue society” [82]. To verify this potential relationship, we also examined whether Living a Calling alleviates the effects of PTSD from violence victimization by students on the self-destructive behavior and turnover intention among teachers brought about by social interaction anxiety and psychological burnout.

### 1.4. Study Model and Hypotheses

**Figure 1:**
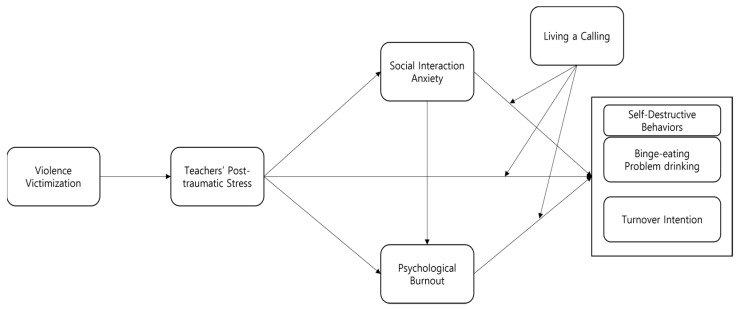
Study model.

Based on the above-mentioned relevant theories and the literature, this study focused on shedding light on the structural relationship between PTSD from violence victimization by students and basic education teachers’ social interaction anxiety, psychological burnout, self-destructive behaviors, and turnover intention, and on identifying whether Living a Calling will alleviate this structural relationship and potentially be a useful therapeutic intervention for these teachers. To this end, we investigated the mediating effects in the relationship between PTSD from violence victimization by students and teachers’ self-destructive behaviors and turnover intention and the moderating effects of Living a Calling (Figure 1). First, in contrast to previous studies that set either social interaction anxiety or psychological burnout as the criterion variable to predict self-destructive behaviors (specifically, binge-eating and problem drinking) or turnover intention [31,34,64], this study included PTSD from violence victimization by students, social interaction anxiety, psychological burnout, and Living a Calling in the study model. Teachers’ psychological problems (e.g., social interaction anxiety and psychological burnout) correlated with a greater percentage of self-destructive behavior and turnover intention compared to other variables (e.g., job stress, job satisfaction, and organizational commitment) [5,37,83], and whether psychological problems would alter behavior was also considered [16]. Based on the AET, we hypothesized that PTSD and psychological problems from violence victimization by students (social interaction anxiety and psychological burnout) precede self-destructive behaviors and turnover intention. Furthermore, based on the AET (cognition → emotion → attitude→ behavior) and the findings of Pietarinen et al. [54], Maslach et al. [20], Lee [34], and Lee and Cho [5], we hypothesized that social interaction anxiety and psychological burnout would mediate the relationship of PTSD from violence victimization by students with teachers’ self-destructive behaviors and turnover intention. A qualitative study that explored the causes of stress among teachers [84] pinpointed PTSD due to violence victimization by students as the cause of teachers’ social, psychological and emotional anxiety and burnout, and Koo and Kim [32] and Kim [85] reported that teachers who have experienced frustration in their role due to infringed rights in school began to doubt their aptitude for their occupation and felt anxiety and burnout.

Past studies on school violence primarily focused on students, with only a handful of studies examining teachers [5,75]. The scope of research on school violence involving teachers has been limited, and the effects of PTSD from school violence on teaching performance and turnover intention have been rarely studied. In this study, we attempted to expand the scope of the definition of victims of school violence to teachers and to understand the structural association between PTSD from school violence victimization and psychological burnout, turnover intention, and problem drinking, to gain a multilateral understanding of the influence of school violence on school faculty. Findings that support this structural relationship would be clinically significant in shedding light on the appropriate use of psychosocial interventions to address social interaction anxiety and psychological burnout among schoolteachers. We set the following hypotheses.

#### Study Hypotheses

**Hypothesis 1 (H1).**  *Social interaction anxiety will mediate the relationship between PTSD from violence victimization and self-destructive behaviors and turnover intention in elementary, middle, and high school teachers.*

**Hypothesis 2 (H2).**  *Psychological burnout will mediate the relationship between PTSD from violence victimization and self-destructive behaviors and turnover intention in elementary, middle, and high school teachers.*

**Hypothesis 3 (H3).**  *Social interaction anxiety and psychological burnout will dually mediate the relationship between PTSD from violence victimization and self-destructive behaviors and turnover intention in elementary, middle, and high school teachers.*

In addition, on the basis of the Theory of Calling by Duffy, Allan, and Bott [79] and the Work as a Calling Model by Duffy et al. [81], Living a Calling was described as a moderator. Accordingly, we set the following hypothesis.

**Hypothesis 4 (H4).**  *Living a Calling will moderate the relationships between the variables of PTSD from violence victimization, social interaction anxiety, psychological burnout, self-destructive behaviors, and turnover intention in elementary, middle, and high school teachers.*

Hypotheses 1–4 suggest a moderated mediating effect [86]. In other words, teachers’ Living a Calling is predicted to moderate the direct relationship between social interaction anxiety, psychological burnout, self-destructive behaviors, and turnover intention, so the indirect relationships between PTSD from violence victimization, the mediating variables (social interaction anxiety and psychological burnout), and the dependent variables (self-destructive behaviors and turnover intention) are also predicted to be affected by the variable of Living a Calling. Previously mentioned study findings [79,81] and De Wet’s [87] argument regarding teachers’ violence victimization support these predictions. We set the following hypothesis regarding the conditional indirect effects of moderated mediation.

**Hypothesis 5 (H5).**  *The effects of PTSD from violence victimization on self-destructive behaviors and turnover intention through the mediation of social interaction anxiety and psychological burnout among elementary, middle, and high school teachers will be alleviated by the factor of Living a Calling. Specifically, the higher the perceived Calling among teachers, the more attenuation there will be in its indirect effects.*

## 2. Materials and Methods

### 2.1. Study Participants and Procedure

This study included elementary, middle, and high school teachers, specifically, current teachers, with symptoms of PTSD consequent to violence victimization by students. The study participants were sampled from current teachers nationwide in Korea. The recruitment announcement was posted on the Mind Healing Center, and other online communities in which most teachers in Korea were registered. Additionally, snowball sampling was employed through an acquaintance who is a teacher. The online questionnaire (Google questionnaire) took approximately 15 min to complete, and all participants who completed the questionnaire were compensated with a Gifticon (gift service app). Data were collected for 22 days, from 2 September to 24 September 2021. A total of 450 elementary, middle, and high school teachers completed the questionnaire; after excluding 30 questionnaires with invalid responses, data from 420 teachers were included in the final analysis. Statistical power was computed prior to the analysis using the G*Power3 software. For eight measurement variables, the minimum sample size was 120; since our sample comprised 420 individuals, our analysis has a high statistical power [88]. This study was approved by the Institutional Review Board (IRB) of Yonsei University to ensure its ethical conduct and scientific validity and reliability (IRB No. Yonsei-7001988-202108-HR-1288-03).

Regarding the demographics of the 420 participants, 207 were female (49.3%), and 213 were male (50.7%). The ratio of male to female teachers in this study is similar to that of the population. Their ages were 20–29 years (n = 22, 5.2%), 30–39 years (n = 89, 21.2%), 40–49 years (n = 159, 37.9%), and ≥50 years (n = 150, 35.7%); lengths of teaching career were <5 years (n = 39, 9.3%), 5–9 years (n = 69, 16.4%), 10–14 years (n = 85, 20.2%), 15–19 years (n = 74, 17.6%), and ≥20 years (n = 153, 36.4%). Of the total sample, 200 (47.6%) participants were homeroom teachers, and the rest were not (52.4%). Further, 131 (31.2%) participants were Department Heads, whereas 289 (68.8%) participants were regular teachers. School sizes were <15 classes (n = 107, 25.5%), 15–29 classes (n = 190, 45.2%), and ≥30 classes (n = 123, 29.3%). School levels were elementary school (n = 146, 34.8%), middle school (n = 123, 29%), and high school (n = 151, 36.0%). A total of 286 (68.1%) participants worked in a public school, whereas 134 (31.9%) participants worked in a private school. The types of employment included lecturers (n = 33, 7.9%), short-term teachers (n = 88, 21.0%), and regular full-time teachers (n = 299, 71.2%). The school regions were Seoul Special City (n = 69, 16.4%), small- and medium-sized cities (n = 290, 69.0%), and rural regions (n = 61, 14.5%).

### 2.2. Instruments

#### 2.2.1. Teacher Violence Victimization Experience Checklist (TVVEC)

We used the TVVEC developed by Wilson, Douglas, and Lyon [6] and adapted and validated by Lee and Cho [5] and Choi [47] to assess teachers’ violence victimization experiences. The TVVEC consists of 15 items—5 items for covert violence (e.g., rude and innocuous gestures aimed at annoying or threatening someone); 6 items for overt violence (e.g., “threatening” someone as if inflicting physical violence without the use of a weapon); and 4 items for other violence (e.g., damaging personal possessions). Each item is rated on a five-point Likert scale (0 = never, 4 = frequently), and a higher score indicates more frequent violence victimization experiences. The factor loadings were 0.500 or higher for covert violence, overt violence, and other violence in a previous study [6], and the factor loadings were higher than 0.500 in the confirmatory factor analysis (CFA) [5]. The TVVEC score correlated significantly with a mental health problem scale and psychological well-being [89]. In addition, the internal consistency of the scale (Cronbach’s α) was 0.930 in a previous study [47]. In the present study, the TVVEC accurately measured the latent variables (factor loading 0.731–0.913). The internal consistency (Cronbach’s α) of the entire scale was 0.917, and those for the covert violence, overt violence, and other violence subscales were 0.919, 0.908, and 0.777, respectively.

#### 2.2.2. Impact of Event Scale Revised Korean Version (IES-R-K)

PTSD symptoms caused by infringed teachers’ rights among the participants were assessed using the IES-R-K, a Korean-validated version [90] of the revised [91] Impact of Event Scale [92]. The IES-R-K consists of 22 items, including 6 items for hyperarousal (e.g., I felt sensitive and angry since the event); 6 items for avoidance (e.g., I tried to avoid thinking about the event because I get confused every time I think about it or it pops up in my head); 5 items for intrusions (e.g., something that reminds me of the event re-aroused the feelings I had at the time); and 5 items for a sleep disorder and emotional paralysis and dissociation (e.g., I had trouble staying asleep). Each item is rated on a five-point Likert scale (1 = strongly disagree, 5 = strongly agree), and a higher score indicates greater severity of PTSD symptoms. The validity of the hyperarousal, avoidance, intrusions, sleep disorder, and emotional paralysis and dissociation subscales of the IES-R-K was established via CFA in previous studies (β = 0.940–0.944) [90,93], and PTSD was significantly correlated with optimism, post-traumatic growth, drinking problems, maladaptive coping, and active coping [94]. Furthermore, Eun et al. [90] reported an internal consistency (Cronbach’s α) of 0.830. In the present study, the IES-R-K (factor loading 0.876–0.924) measured the latent variables with an acceptable level of accuracy; the internal consistency (Cronbach’s α) of the entire scale was 0.979, and those for the hyperarousal, avoidance, intrusions, sleep disorder and emotional paralysis and dissociation subscales were 0.962, 0.950, 0.964, and 0.925, respectively.

#### 2.2.3. Social Interaction Anxiety Scale (SIAS)

Social interaction anxiety among the teachers was measured using the SIAS developed by Mattick and Clarke [36] and adapted and validated by Park and Chung [35] and Kim [95]. The SIAS consists of 19 items (e.g., I have trouble making eye contact with others, I have trouble hanging out with my friends) and measures perceived anxiety during social interactions or due to others’ judgment and experiences with others. Each item is rated on a five-point Likert scale (1 = strongly disagree, 5 = strongly agree), and a higher score indicates greater social interaction anxiety. The validity of the SIAS was evaluated as a single-factor scale in a previous study, and the factor loading ranged from 0.672 to 0.700 [35]. Social interaction anxiety was significantly correlated with internalized embarrassment, perceived social support, ambivalent emotional expression, defensive ambivalence, and relational ambivalence. The internal consistency (Cronbach’s α) was 0.950 in the study by Park and Chung [35]. In the present study, the SIAS measured the latent variable at an acceptable level of accuracy (factor loading 0.752–0.951), and the internal consistency of the entire scale (Cronbach’s α) was 0.949.

#### 2.2.4. Maslach Burnout Inventory (MBI)

Psychological burnout among the teachers was assessed using the MBI developed by Maslach and Jackson [53] and adapted and validated by Kim [51], Kang [50], and Yoo and Park [52]. The MBI consists of nine items for emotional exhaustion (e.g., My mind and body are exhausted from work); five items for depersonalization (e.g., I feel like I treat some students as non-living objects), and eight items for reduced personal accomplishment (e.g., I can easily understand what students think and feel) for a total of 22 items (items 4, 7, 9, 12, 17, 18, 19, 21 are reverse-worded). Each item is rated on a six-point Likert scale (0 = never, 6 = every day), and a higher score indicates a greater degree of psychological burnout. The MBI was found to have a factor loading of 0.830–0.870 for its three subscales [23,51,52], and it was significantly correlated with emotional labor superficial behaviors, emotional labor internal behaviors, emotional dissonance, and satisfaction of basic psychological needs. In the study by Kang [50], the reliability of the subscales was 0.857 for emotional exhaustion, 0.639 for depersonalization, and 0.774 for reduced personal accomplishment. In the present study, the MBI measured the latent variable at an acceptable level of accuracy (factor loading 0.673–0.822). The internal consistency (Cronbach’s α) of the entire scale was 0.918, and those for the emotional exhaustion, depersonalization, and reduced personal accomplishment subscales were 0.915, 0.823, and 0.893, respectively.

#### 2.2.5. Self-Destructive Behaviors

Binge-eating (Eating Disorder Inventory-2 or EDI-2)

Symptoms of eating disorders such as anorexia nervosa and bulimia nervosa among the teachers were measured using seven items developed by Garner and Olmsted [96] and Garner [97], and adapted and validated by Cho [98], Lee [99], and Heo [100]. The EDI-2 consists of eight subscales and three scales, but in the present study, we used the seven items adapted and validated by Cho [98] and Heo [100] (e.g., I eat when I am hurt). Each item is rated on a six-point Likert scale (1 = not at all, 6 = always), and a higher score indicates more frequent binge-eating behaviors. Regarding validity, the EDI-2 was evaluated as a one-factor scale, and the factor loading was higher than 0.500; CFA also confirmed validity [96,98,100], and binge-eating was significantly correlated with clear emotional recognition and impulsivity. In the study by Heo [100], the reliability of the scale was 0.860. In the present study, the EDI-2 measured the latent variable at an acceptable level of accuracy (factor loading 0.631–0.915), and the internal consistency of the entire scale (Cronbach’s α) was 0.907.

Alcohol Use Disorders Identification Test (AUDIT-K)

We used 10 items from the AUDIT-K developed by Allen et al. [101] and adapted and validated by Lee et al. [102] to measure symptoms of problem drinking (e.g., How often do you drink? How often do you drink at least one bottle of soju per sitting?) among the teachers. Each item was rated on a five-point Likert scale (1 = I don’t drink at all, 6 = every day), and a higher score indicates more frequent problem-drinking behaviors. Items 9 and 10 were measured using a three-point Likert scale (1 = never, 3 = yes in the past year). Regarding validity, the AUDIT-K was evaluated as a one-factor scale, and the factor loading ranged from 0.500 to 0.900 [102]. Problem drinking was significantly correlated with optimism and maladaptive coping [94]. Lee et al. [102] reported an internal consistency (Cronbach’s α) of 0.920. In the present study, AUDIT-K measured the latent variable at an acceptable level of accuracy (factor loading 0.764–0.899), and the internal consistency (Cronbach’s α) was 0.894.

#### 2.2.6. Turnover Intention (TI-K) Scale

Turnover intentions among the teachers were measured using the Turnover Intention Scale developed by Landau and Hammer [103] and adapted and validated by Lee et al. [104]. The scale consists of three items (e.g., I am actively looking for another job, I will leave my job as soon as I find a better one). Each item is rated on a five-point Likert scale (1 = strongly disagree, 5 = strongly agree), and a higher score indicates greater turnover intention. The validity of the TI-K was evaluated as a one-factor scale, and the factor loading was higher than 0.500 [103]. The turnover intention was significantly correlated with the utilization of systems, a family-friendly culture, work–life conflict, and emotional organizational commitment [104]. The internal consistency (Cronbach’s α) was 0.900 in the study by Lee et al. [104]. In the present study, TI-K measured the latent variable at an acceptable level of accuracy (factor loading 0.733–0.939), and the internal consistency (Cronbach’s α) was 0.857.

#### 2.2.7. Living a Calling Scale (LCS)

Living a Calling among the teachers was measured using the LCS developed by Duffy et al. [79] and adapted and validated by Jang and Lee [70]. The LCS consists of six items (e.g., I often have the opportunities to live my calling, I am living my calling in my current job, etc.). Each item is rated on a seven-point Likert scale (1 = strongly disagree, 7 = strongly agree), and a higher score indicates a greater degree of Living a Calling. The validity of the LCS was evaluated as a one-factor scale, and the factor loading ranged from 0.940 to 0.980. Living a Calling was significantly correlated with Perceiving a Calling, meaning of work, meaning in life, job satisfaction, and life satisfaction [70]. The internal consistency (Cronbach’s α) was 0.970 in the study by Jang and Lee [70]. In the present study, the LCS measured the latent variable at an acceptable level of accuracy (factor loading 0.705–0.960), and the internal consistency (Cronbach’s α) was 0.962.

### 2.3. Item Parceling

As the items that measure “anxiety” in the SIAS belong to one factor, we anticipated the possibility of measurement error, estimation error, and problems with the fit indices of the overall study model [43,105,106,107]. Hence, we performed item parceling to lower the ratio of total cases to measurement values to evaluate the factor structure of the measurement model and the structural causality of the structural model [105,106]. Three observed items are considered ideal (just identified) to stably measure a single latent variable [107], so we first hypothesized a one-factor model for “anxiety” in the SIAS using maximum likelihood and performed unrotated exploratory factor analysis. Then, we parceled the observed items with the highest absolute value of factor loading to the lowest absolute value of factor loading to produce four item parcels with similar loadings to those of the latent variable.

### 2.4. Data Analysis

In this study, the data collected were empirically analyzed using SPSS 26.0, Amos 28.0, and Process Macro 4.0. The empirical analysis was divided into a measurement model and a structural model, and the specific protocol was as follows: first, the demographic characteristics of the teachers were analyzed with frequency analysis using the SPSS 26.0 software, and the reliability and validity of the instruments used for the measurement model were evaluated. Second, since the data were collected from a single source, the common method bias was tested using Harman’s One Factor Test. Third, the mean, standard deviations of the study variables, correlations among study variables, and discriminant validity were analyzed. Finally, the study hypotheses were tested based on the results of the measurement model by performing structural equation modeling (SEM); we investigated whether PTSD from violence victimization by students influences self-destructive behaviors and turnover intention when mediated by social interaction anxiety and psychological burnout and whether this influence is alleviated by Living a Calling, using Process Macro Model 89. To verify the sequential mediation model and explore the contextual factors of Living a Calling, we used the Amos 28.0 software for confirmatory analysis of the study model and Process Macro to examine the moderating effects [105,108]. Specifically, Amos was used for theoretical analysis using the Covariance-Based Structural Equation Model (CB-SEM) algorithm, and Process Macro was used for theoretical exploration through ordinary least squares (OLS) regression using the partial least squares method, primarily to explore contextual factors (moderating effect) [109]. Thus, unlike other tools for mediation analysis, Process Macro enabled the testing of a multi-mediation model reflecting measurement errors and the statistical analysis of each mediating effect [86]. One drawback of regression analysis is the high risk of biased effect estimates compared to SEM due to random measurement error [110]. However, in the comparative study by Hayes et al. [111], no differences were noted in the estimated coefficients (even in a small sample) between the OLS regression method and SEM. Although the standard error differed, this is expected because sample variance estimation was performed based on different statistical assumptions between the OLS and ML (maximum likelihood) and is, thus, negligible. The main focus of this study was to explore the mediating effects between the variables of PTSD from violence victimization, social interaction anxiety, psychological burnout, self-destructive behaviors, and turnover intention, as well as the moderated mediating effects of Living a Calling among teachers. We tested our hypotheses using Process Macro.

## 3. Results

### 3.1. Descriptive Statistics

The means and standard deviations of latent variables, correlations, and the discriminant validity of latent variables are presented in Table 1. CFA and discriminant validity testing were performed to determine whether the observed items accurately explain the latent variable and to verify the discriminatory power of latent variables.

First, the absolute values of skewness and kurtosis of all latent variables did not exceed 2 and 7, respectively, confirming that the data are normally distributed [112,113]. Thus, the assumption of normality of maximum likelihood, a method of testing a structural equation model and identifying causal inference, was deemed satisfactory. The maximum likelihood method is a more realistic and non-biased method of estimation in which the observed is fixed on the assumption that the collected data are from a sample and not a population, and optimized observed values are computed to produce estimates identical to the actual parameter. In this way, the estimated values move closer to the actual parameters with increasing sample size, and the computed estimates have similar efficiencies.

Second, CFA was performed to test whether the observed items accurately reflect and explain the corresponding latent variables (teacher violence victimization experience, PTSD, social interaction anxiety, psychological burnout, binge-eating, turnover intention, and problem drinking). The measurement model was found to fit the data (*x^2^* = 1007.060, df = 521, CMIN/DF = 1.933, goodness-of-fit index [GFI] = 0.874, adjusted goodness-of-fit index [AGFI] = 0.848, comparative fit index [CFI] = 0.962, normed fit index [NFI] = 0.925, incremental fit index [IFI] = 0.962, Tucker–Lewis index [TLI] = 0.956, root mean square error of approximation [RMSEA] = 0.047, root mean square residual [RMR] = 0.063). In addition, all observed items were significantly loaded onto their corresponding latent variables (factor loading β = 0.631–0.960, *p* < 0.001). Construct validity, content validity, and convergent validity were established for all latent variables [108]. However, since we collected data through a questionnaire, common method bias cannot be ruled out. To resolve this issue, we performed a post hoc Harman’s One Factor Test. EFA was performed for a one-factor model, and the model explained 27.20% of the total variance. CFA also showed that the model with one latent variable for all observed variables had a markedly lower fit than the measurement model (*x^2^* = 7541.976, df = 549, CMIN/DF = 13.738, GFI = 0.442, AGFI = 0.360, CFI = 0.451, NFI = 0.435, IFI = 0.453, TLI = 0.405, RMSEA = 0.174, RMR = 0.248). Therefore, we determined that the common method bias of the data collected for our latent variables is not serious enough to influence our study results [114].

Third, correlations among the latent variables and independence (discriminant validity) as study variables (teacher violence victimization experience, PTSD, social interaction anxiety, psychological burnout, binge-eating, turnover intention, and problem drinking) were analyzed to describe the strength of association among the constructs and establish discriminant validity. Correlation and discriminant validity testing measure the strength of correlations among the constructs of the latent variables. They also show the associations among the constructs of the latent variable prior to testing the hypotheses, in addition to establishing the discriminatory power of each study variable. The presence of association among the constructs of latent variables is determined based on the strength and significance of their correlations, and the variables are divided into independent, mediator, dependent, and moderator variables. Thus, correlation and discriminant validity testing are essential before testing all hypotheses [86]. In the correlation analysis, significant correlations were observed between all constructs: teacher violence victimization experience, PTSD, social interaction anxiety, psychological burnout, binge-eating, turnover intention, and problem drinking. The correlations among the study variables are shown in Table 1. Violence victimization was positively correlated with PTSD (r = 0.56), social interaction anxiety (r = 0.34), psychological burnout (r = 0.38), turnover intention (r = 0.32), binge-eating (r = 0.31), and problem drinking (r = 0.25) but negatively correlated with Living a Calling (r = −0.17). PTSD was positively correlated with social interaction anxiety (r = 0.47), psychological burnout (r = 0.42), turnover intention (r = 0.26), binge-eating (r = 0.30), and problem drinking (r = 0.15), and negatively correlated with Living a Calling (r = −0.13). Social interaction anxiety was positively correlated with psychological burnout (r = 0.51), turnover intention (r = 0.25), binge-eating (r = 0.36), and problem drinking (r = 0.24), and negatively correlated with Living a Calling (r = −0.21). Psychological burnout was positively correlated with turnover intention (r = 0.41), binge-eating (r = 0.43), and problem drinking (0.33), and negatively correlated with Living a Calling (r = −0.56). Living a Calling was negatively correlated with turnover intention (r = −0.38), binge-eating (r = −0.23), and problem drinking (r = −0.11).

Fourth, discriminant validity was tested by comparing the average variance extracted (AVE) of latent variables and the square of correlation coefficients for the latent variables, where discriminant validity is deemed established if the AVE is greater than the R^2^ [108]. As shown in Table 1, the largest correlation coefficient was 0.56 (violence victimization and PTSD), and the coefficient of determination (R^2^) was 0.31 (0.56 × 0.56); the smallest AVE value was 0.67, which is greater than 0.31, so the latent variables are deemed to have discriminant validity. Composite reliability (ComR) and AVE were calculated and interpreted per the Fornell and Larcker [108] criterion. AVE is calculated by dividing the sum of the square of standardized factor loadings by the sum of the square of standardized factor loadings of the observed variables and the sum of the error variance of the observed variables [108,115]. The construct reliability or ConR of latent variables is calculated by dividing the sum of the square of standardized factor loadings of the observed variables by the sum of the square of standardized factor loadings and the sum of error variance. In this study, the ConR values were as follows: violence victimization (0.896), PTSD (0.957), social interaction anxiety (0.955), psychological burnout (0.855), binge-eating (0.927), turnover intention (0.920), problem drinking (0.939), and Living a Calling (0.970). The criterion for AVE (≥0.500) and ConR (≥0.700) were both met [108].

### 3.2. Evaluation of the Structural Model and Direct Effects

**Figure 2:**
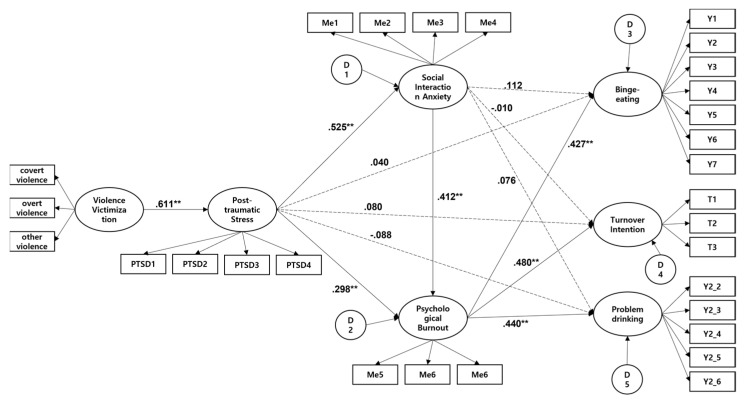
A structural model for PTSD from violence victimization, social interaction anxiety, psychological burnout, self-destructive behaviors, and turnover intention. Note. *n* = 420. ** *p* < 0.01.

The structural model fit the data (*x^2^* = 836.879, df = 358, CMIN/DF = 2.338, GFI = 0.873, AGFI = 0.846, CFI = 0.948, NFI = 0.912, IFI = 0.948, TLI = 0.941, RMSEA = 0.057, RMR = 0.063). Although most path coefficients for direct effects were significant, the direct path from social interaction anxiety to binge-eating, turnover intention, and problem drinking were not significant. The study model explained 23.6% of binge-eating, 22.9% of turnover intention, and 12.7% of problem drinking. Martens [116] proposed that the study model should be compared to an alternative model to reduce respondents’ confirmation bias. In particular, a nested model, which pairs the same number of latent variables but in different ways, allows the identification of the optimal model through a direct comparison between a more parsimonious model and a complex model [115]. Therefore, we set the model that excluded insignificant paths as the revised model and tested its fit. The results confirmed that the model fits the data. Specifically, the fit of the alternative model was *x^2^* = 1110.907, df = 369, CMIN/DF = 3.011, GFI = 0.827, AGFI = 0.796, CFI = 0.919, NFI = 0.884, IFI = 0.919, TLI = 0.911, RMSEA = 0.069, RMR = 0.068. A chi-square test showed that the fit of the study model and alternative model did not differ: Δ*x^2^* = (11)274.028, *p* > 0.05. Thus, the more parsimonious model was adopted as the final model, presented in Figure 2.

In the final model, PTSD from violence victimization positively affected social interaction anxiety and psychological burnout (β = 0.575, *p* < 0.001, β = 0.298, *p* < 0.001). This means that the level of social interaction anxiety and psychological burnout increases with increasing PTSD from violence victimization. Further, PTSD from violence victimization and social interaction anxiety did not significantly affect binge-eating, turnover intention, and problem drinking, whereas psychological burnout significantly affected these variables (β = 0.427, *p* < 0.001; β = 0.480, *p* < 0.001; β = 0.440, *p* < 0.001). Finally, social interaction anxiety significantly affected psychological burnout (β = 0.412, *p* < 0.001).

### 3.3. Evaluation of the Mediation Model

In this study, the hypothesis for the mediating effect was tested using the CB-SEM in Amos 28.0. The structural relationships between the independent variable (PTSD from violence victimization); mediators (social interaction anxiety and psychological burnout); and dependent variables (turnover intention and self-destructive behaviors of binge-eating and problem drinking) were examined, and SEM was performed to analyze whether PTSD from violence victimization affected turnover intention and self-destructive behaviors through the mediation of social interaction anxiety and psychological burnout. Further, the mediating effects (dual mediating effect) of social interaction anxiety and psychological burnout were analyzed using the bootstrapping bias-corrected method. This method determines and estimates the validity of the indirect effects of a mediating model using the upper and lower limits of the confidence interval (modern statistical estimation), where the asymmetry of the bootstrap estimates is more rigorously reflected than traditional statistical estimation (*p*-value: *p* < 0.05) [86,115]. We chose this method because it can produce more accurate results when the sample distribution of the estimates does not follow a normal distribution [115]. With bootstrapping estimation, the 95% confidence interval (CI) (between the upper and lower limit) must not include 0 for the indirect effect to be statistically significant [86,115]. We performed SEM based on the CI-based mediation estimation proposed by Hayes [86]; the results of the hypothesis testing were as follows: the mediation model fits the data (*x*^2^ = 836.879, df = 358, CMIN/DF = 2.338, GFI = 0.873, AGFI = 0.846, CFI = 0.948, NFI = 0.912, IFI = 0.948, TLI = 0.941, RMSEA = 0.057, RMR = 0.063). As shown in Figure 2, PTSD from violence victimization had a positive effect on social interaction anxiety (β = 0.379, *p* < 0.001) and psychological burnout (β = 0.275, *p* < 0.001), and PTSD from violence victimization had a positive effect on psychological burnout through the mediation of social interaction anxiety (β = 0.200, *p* < 0.001); however, PTSD from violence victimization did not affect binge-eating (β = 0.052, *p* > 0.05), turnover intention (β = −0.004, *p* > 0.05), and problem drinking (β = 0.020, *p* > 0.05) through the mediation of social interaction anxiety. PTSD from violence victimization had a positive effect on binge-eating (β = 0.112, *p* < 0.001), turnover intention (β = −0.109, *p* < 0.001), and problem drinking (β = 0.066, *p* < 0.05) through the mediation of psychological burnout.

We examined the dual mediating effects of social interaction anxiety and psychological burnout on the relationships between PTSD from violence victimization and binge-eating, turnover intention, and problem drinking. The results showed that PTSD from violence victimization indirectly affected psychological burnout through social interaction anxiety, while social interaction anxiety indirectly affected binge-eating, turnover intention, and problem drinking through psychological burnout (Table 2). Social interaction anxiety mediated the causal relationship between PTSD from violence victimization and psychological burnout. Further, PTSD from violence victimization affected binge-eating, turnover intention and problem drinking through the mediation of social interaction anxiety and psychological burnout.

### 3.4. Evaluation of the Moderated Mediating Effect

We evaluated whether Living a Calling moderated the relationship of PTSD from violence victimization with binge-eating, turnover intention, and problem drinking, whether Living a Calling moderated the effects of PTSD from violence victimization on binge-eating, turnover intention, and problem drinking through the mediation of social interaction anxiety and psychological burnout. First, the variables were mean-centered to minimize multicollinearity and eliminate errors in interpreting the moderating effects [117]. The moderated mediating model that combines the mediation model with the moderator was analyzed using Model 89 on Process Macro 4.0. This model predicts that a moderator moderates the causal structural relationship between independent variable → mediator, as follows: (1) →mediator (2) →mediator (3) →dependent variable, where the indirect effects increase with the increasing degree of the moderator [86]. In the present study, the moderator (Living a Calling) is a continuous variable; hence, we analyzed whether the mediating effects increased or decreased depending on the level of Living a Calling. For example, PTSD (Mediator) can mediate the relationship of violence victimization with binge-eating, turnover intention, problem drinking, and PTSD (Mediator1); social interaction anxiety (Mediator 2), and psychological burnout (Mediator 3) can sequentially mediate the relationship of violence victimization with binge-eating, turnover intention, and problem drinking. In this case, the mediating effects of PTSD from violence victimization  → social interaction anxiety  → psychological burnout  → binge-eating, turnover intention, and problem drinking will vary depending on the degree of the moderator (Living a Calling). This means that the mediating effects will be high with a low level of Living a Calling but lower with a high level of Living a Calling. To examine whether Living a Calling has a moderated mediating effect, we used the Process Macro protocol proposed by Hayes [86]. First, the indirect effects of PTSD from teacher violence victimization by students on turnover intention were alleviated by Living a Calling (Table 3). This suggests that PTSD among teachers increases with increasing violence victimization experience, which in turn increases turnover intention (mediating effect), and that such mediating effect declines with increasing experiences of Living a Calling (moderating effect). Moreover, contrary to our prediction, the indirect effects of PTSD from teacher violence victimization by students on turnover intention through social interaction anxiety increased with an increasing level of Living a Calling. That is, PTSD from violence victimization did not indirectly affect turnover intention through social interaction anxiety, but indirect effects were observed with a high level of Living a Calling. This may be attributable to the possibility that teachers who perceive themselves to be Living a Calling experience more social interaction anxiety compared to those who do not, and that the former group wishes to leave their jobs due to psychological conflicts pertaining to vocational calling, reputation, and role ambiguity (Table 4).

However, Living a Calling did not have a significant moderated mediating effect on the relationship between PTSD from violence victimization and turnover intention through a dual mediation of social interaction anxiety and psychological burnout. Furthermore, Living a Calling did not have a significant moderated mediating effect on: (1) The effects of PTSD from violence victimization on binge-eating and problem drinking through the mediation of social interaction anxiety (2) The effects on binge-eating and problem drinking through the mediation of psychological burnout; and (3) The effects on binge-eating and problem drinking through a dual mediation of social interaction anxiety and psychological burnout (Table 3).

As shown in Table 5, Living a Calling had a significant moderated mediating effect on the effects of violence victimization on turnover intention through the mediation of PTSD (95% CI lower limit = −0.2372, upper limit = −0.0821). Specifically, the mediating effect (β) was 0.2653 with a low level of Living a Calling (lower limit = 0.1172, upper limit = 0.4245). The mediating effect (β) was 0.0684 with a moderate level of Living a Calling (95% CI lower limit = −0.0629, upper limit = 0.1586) and was insignificant; the mediating effect (β) further decreased to –0.1751 with the highest level of Living a Calling (95% CI lower limit = −0.3290, upper limit = −0.0167). Thus, Living a Calling had a significant moderated mediating effect on the relationship between violence victimization and turnover intention through PTSD.

As shown in Table 5, Living a Calling had a significant moderated mediating effect on the effects of violence victimization on turnover intention through the mediation of PTSD and social interaction anxiety (95% CI lower limit = 0.0217, upper limit = 0.0878). Specifically, the mediating effect (β) was –0.0570 with a low level of Living a Calling (lower limit = 0.1221, upper limit = 0.0082), and the mediating effect (β) was 0.0184 with a moderate level of Living a Calling (95% CI lower limit = −0.0289, upper limit = 0.0651) and an insignificant level of Living a Calling; however, contradictory to our hypothesis, the mediating effect (β) increased to 0.0391 with the highest level of Living a Calling (95% CI lower limit = 0.0302, upper limit = 0.1584). Thus, Living a Calling had a significant moderated mediating effect on the relationship between violence victimization and turnover intention through PTSD and social interaction anxiety, but the direction of the effects differed from our hypothesis.

## 4. Discussion

The purpose of this study was to investigate the associations between violence victimization, the consequent PTSD, self-destructive behaviors, and turnover intention, besides examining the structural relationships with mediators of these associations and moderators of the mediation paths among elementary, middle, and high school teachers based on relevant theories and the literature. We first examined whether PTSD from violence victimization affects self-destructive behaviors and turnover intention by mediating social interaction anxiety and psychological burnout based on the AET [16]. Simultaneously, we analyzed whether Living a Calling moderated this relationship based on the Theory of Calling by Duffy et al. [79] and Work as a Calling by Duffy et al. [81]. Our discussion is based on our hypotheses with reference to relevant theories and the existing literature.

First, PTSD from violence victimization may positively affect self-destructive behaviors and turnover intention through psychological burnout in teachers. This suggests that teachers who have been victimized by student violence and consequently develop severe PTSD, exhaust their physical and emotional resources, distance themselves from students, or have a decreased sense of accomplishment and productivity; consequently, they may engage in self-harming behaviors, such as binge-eating and problem drinking, or they may consider leaving their jobs. These findings are partially in line with previous findings showing the mediating effect of burnout on the relationship between job stress and turnover intention in childcare teachers [118], the mediating effects of job stress and burnout on the relationship between mental violence victimization and turnover intention among social workers [119], and the correlations between job distress, binge-eating, and problem drinking among physicians [120]. Particularly, psychological burnout was found to completely mediate the effects of PTSD from violence victimization on self-destructive behaviors and turnover intention among the teachers. This means that teachers who experience violence and consequently develop PTSD are subject to increased psychological burnout in their jobs, leading to self-destructive behaviors, such as binge-eating and problem drinking, or an increase in turnover intention.

However, social interaction anxiety did not significantly mediate the relationship between PTSD from violence victimization and self-destructive behaviors and turnover intention among the teachers. That is, social interaction anxiety cannot explain the reason for the association between PTSD from violence victimization and self-destructive behaviors and turnover intention. Hence, psychological burnout should be focused on, rather than social interaction anxiety, regarding teachers who have suffered from student violence. Moreover, the fact that social interaction anxiety is not a mediator in this relationship suggests that another mediator may be involved. Past studies have investigated job satisfaction in relation to workplace violence victimization and turnover intention [121,122,123,124]; thus, subsequent studies could explore whether job satisfaction and other factors mediate the relationship between PTSD from violence victimization and self-destructive behaviors and turnover intention.

Second, PTSD from violence victimization indirectly affected psychological burnout through social interaction anxiety, and social interaction anxiety indirectly affected binge-eating, problem drinking, and turnover intention through psychological burnout among the teachers. Specifically, social interaction anxiety mediated the relationship between PTSD from violence victimization and psychological burnout; social interaction anxiety and psychological burnout dually mediated the relationship between PTSD from violence victimization and binge-eating, problem drinking, and turnover intention. These results are partially in line with the findings of Kachadourian et al. [125], who examined the relationship between trauma, PTSD, binge-eating, and problem drinking among adults who had experienced a traumatic event, and Paltell et al. [126], who examined the moderating effects of the quality of relationships on the relationship between PTSD and problem drinking in undergraduates. This may suggest that when teachers with severe PTSD from violence victimization feel anxious about not being able to respond appropriately or being ignored in social interactions with other people, they become dull in their work as a teacher (which includes fostering relationships with these individuals), fail to achieve a sense of accomplishment, and exhaust their physical and emotional energy, they may engage in binge-eating, drink alcohol, or consider quitting their jobs as a means of escaping their psychologically burned-out state [127,128]. In addition, these results partially support the AET [16], which describes how situational factors at work or an affective response to an event at work form one’s attitude and alter one’s behaviors (cognition → emotion → attitude → behavior). As the AET argues, PTSD (cognition) may sequentially lead to social interaction anxiety (emotion) and psychological burnout (attitude), ultimately triggering self-destructive behaviors (behavior). However, existing study data on whether turnover intention predicts actual resignation from the job (behavior) are inconsistent [129,130]. Thus, this topic needs further research among teachers with PTSD from violence victimization.

Third, the effects of violence victimization on turnover intention through PTSD among teachers were alleviated by the level of teachers’ Living a Calling. This means that teachers who perceive themselves to be Living a Calling may demonstrate a lower turnover intention than their counterparts with a low level of Living a Calling, even if they develop PTSD from violence by students. The findings are consistent with previous results that the degree of Living a Calling leads to low turnover intention through work engagement [64], and that an increased level of challenging tasks completely mediates the adverse effects on turnover intention [131]. Furthermore, our results are in line with previous research findings that workers with a high sense of Calling and low emotional burnout are less likely to consider leaving their jobs [132], and that these people are highly motivated to be involved in their work and have high internal motivations for work [133]. Our results can be interpreted as follows: teachers who develop PTSD after being exposed to student violence may consider continuing their teaching career challenging. However, having a sense of Calling means that they recognize their Calling and are living their Calling, which would boost their self-esteem in undertaking their duties as a teacher even in challenging situations, and serve as a coping mechanism that motivates them to explore meaning in their teaching career, thereby suppressing any vestige of intending to leave their teaching career. This means that teachers with a high sense of Living a Calling do not overly focus on the negative aspects of their PTSD from violence victimization and instead accept it as an inevitable aspect of their job; hence, they underrate the repercussions of violence victimization and instead focus on other positive outcomes, which may, in turn, lead to low turnover intention.

Our hypothesis on the effects of teachers’ PTSD from violence victimization on self-destructive behaviors and turnover intention through the mediation of social interaction anxiety and psychological burnout was not supported. That is, Living a Calling moderated the direct effect of PTSD on turnover intention but not the indirect effect of PTSD on turnover intention through social interaction anxiety and psychological burnout. As mentioned by Lee and Cho [5], this result highlights the need to pay attention to fear and embarrassment—two types of emotions that mediate the relationship between teachers’ violence victimization and burnout. Due to the Korean culture being characterized by a strict hierarchy between teachers and students, experiencing violence from students and, consequently, suffering from PTSD may intensify teachers’ embarrassment. Considering that self-destructive behaviors, namely binge-eating and problem drinking, at least temporarily alleviate tension and stress despite being unhealthy coping mechanisms, Living a Calling did not curtail social interaction anxiety and psychological burnout caused by PTSD. This suggests that there is another mediator involved in the effects of violence victimization on the turnover intention that is a stronger factor than teachers’ perception of Living a Calling alone. Particularly, interventions targeting teachers’ emotions, social interaction anxiety, and psychological burnout, which serve as mediators, are essential to preventing negative outcomes such as self-destructive behaviors and turnover intention. There is a need for more systematic- and organizational-level interventions than personal-level interventions for the Living a Calling factor.

Finally, the most interesting finding of this study was that Living a Calling reduced the effects of PTSD on turnover intention but had a positive effect on the indirect effects of PTSD on turnover intention in a study model involving social interaction anxiety as a mediator. Specifically, this result was observed in the group with Living a Calling at one standard deviation higher than the average, which partially supports previous findings showing a significant positive direct relationship between Living a Calling and turnover intention [65], and increased anxiety among teachers with high social interaction anxiety due to greater doubts about their work, professional efficacy, goals and future plans, and decision to continue or quit their teaching career [30]. This means that teachers with a higher sense of Living a Calling were subject to greater effects of PTSD from violence victimization on their turnover intention through social interaction anxiety than their counterparts with a lower sense of Living a Calling. That is, the perception of Living a Calling suppressed the link between school violence victimization among teachers and their turnover intention. However, in the presence of social interaction anxiety, turnover intention increased with an increasing sense of Living a Calling. This contradicts our hypothesis and can potentially be attributed to various reasons described below.

First, when PTSD from violence victimization leads to social interaction anxiety among teachers, teachers with a high sense of Living a Calling may experience increased turnover intention as an alternative in order to maintain their Calling. This is due to the nature of Calling as a lifelong progressive task, which is embodied and clarified as one accumulates knowledge and experience in their line of work [61]. Through their Work as a Calling Theory, Duffy et al. [81] argued that Living a Calling can predict both positive and negative life outcomes. This means that Living a Calling can be linked to an attitude of relentlessly seeking better careers and being open to new jobs [64]; hence, teachers with a higher sense of Living a Calling may more actively seek opportunities to move onto another job with a better environment that allows them to concentrate on their work when their current job does not provide this.

In addition, the fact that PTSD from school violence victimization leads to social interaction anxiety suggests that teachers’ role conflicts are involved. Role conflict is defined as the degree to which a member of an organization is expected to play two incompatible roles from their boss or colleagues [134]. Schools are special work environments where teachers are bound to spend much time with their colleagues and students. However, role conflict may occur when teachers who develop PTSD from violence by students are criticized by their colleagues for not fulfilling their duties (e.g., leading students to the correct path and maintaining dignity), as opposed to receiving acceptance and support from colleagues. Particularly, teachers with a high sense of Living a Calling value developing self-esteem, a sense of fulfillment, and duty from their teaching career, recognizing their Calling as a teacher and living their Calling. Thus, these teachers are more likely to perceive a greater degree of role conflict in the organization than their counterparts with a lower sense of Living a Calling. These results are partly in line with the findings of a qualitative study involving workers in Korea [32], where a cynical and performance-centered organizational culture and a lack of support within the organization are reported as barriers to Living a Calling. Another study involving Korean pilots found that a sense of Calling reduced role conflict but mediated the positive effect of role conflict on turnover intention [74]. However, these inferences are made based on past study findings to understand the moderated mediating effect of Living a Calling on turnover intention, and more empirical studies are needed on the topic.

Finally, the reason for the lack of a moderated mediating effect of Living a Calling may be that teachers with an average or lower-than-average sense of Calling value the practical aspects of their work more, as compared with Living a Calling, and consequently, these teachers may focus on saving their reputation. This is contextually in line with a foreign study on healthcare workers [66], where workers with a low sense of Calling developed more organizational attachment in organizations with high job stability, and as a result, these workers had a lower turnover intention than those with a high sense of Calling. Workers with a high sense of Calling view their job and organization as a means to realize their Calling, and thus, become attached to their organization, whereas workers with a low sense of Calling view their job and organization as a means to make a living or achieve other purposes, such as gaining social position [135]. Traditionally, teaching is known as a stable job. Thus, it is possible that PTSD from student violence and the consequent social interaction anxiety will not motivate teachers with a low sense of Calling to quit or move to a different workplace.

## 5. Conclusions

The findings of this study have the following implications. First, this study presents academic and practical implications in that it has focused on teachers as the victims of school violence. In most of the literature, the term “school violence” focuses on “students” as the victims [7]. This is important in terms of providing legal protection to student victims, but it also demonstrates that academic institutions and schools do not pay much attention to teacher victims of violence. With a growing number of cases in which teachers’ rights are violated, teachers are suffering an array of negative effects, including the loss of motivation, reduced concentration, avoidance of meeting with parents, psychological burnout [47], sleep disorders, headaches, eating disorders, and hypertension [48], which undermine their ability to teach and guide students [5]. As shown here, teachers’ experience of being victimized by student violence can adversely impact students as well, but research on teacher victimization through student violence is scarce despite the need to implement measures to address the issue [42]. Studies have only recently begun to examine teachers as victims [7,34,47], but even these studies have mostly involved qualitative research methodologies, posing limitations in gaining a comprehensive understanding of violence victimization among teachers. The present study has broadened the scope of research on school violence by showing that violence victimization among current school teachers may result in their engagement in self-destructive behaviors and turnover intention. We have also shed some light on the psychological mechanisms underlying the associations between social interaction anxiety, psychological burnout, and Living a Calling.

Second, this study presents practical implications for counseling, as it explored violence victimization among teachers in the context of trauma, and discovered the mediating effects of social interaction anxiety and psychological burnout, and the moderating effect of Living a Calling, which appear to be related to the negative consequences of binge-eating, problem drinking, and turnover intention. Being exposed to student violence is a severely traumatic experience that can inflict long-term and serious harm on teachers. A qualitative study on teachers’ violence victimization experiences observed that teachers who were exposed to physical and verbal violence from parents or students suffered serious emotional and relational consequences in their personal and professional lives, including loss of confidence, intensified fear, an inability to deal with the related emotions, hypersensitivity and depression, avoidance of interpersonal relationships, and engagement in harsh language and behaviors [4]. Other studies reported that student violence against teachers led to adverse health outcomes, including burnout, depression, anxiety, and paranoia [46,136], and is associated with negative emotions, a negative perception of fairness in the world [137], and the tendency to leave the teaching profession [138]. We also observed that violence victimization, directly and indirectly, affected self-destructive behaviors such as binge-eating, problem drinking, and turnover intention, consistent with previous findings. One study also reported that the violence victimization experience among teachers is a traumatic event, and these teachers develop serious symptoms compared to those experienced by other individuals having PTSD [139]. Therefore, counseling and treatment are essential for teachers victimized by student violence.

In counseling interventions for PTSD among teachers exposed to student violence, one effective strategy is to promote the subjective interpretation and evaluation of the traumatic experience and the integration of the meaning of trauma with the self-system [7]. Ehlers and Clark [140] stated that the dynamic interactions between intrapersonal factors and environmental factors must be considered before and after the trauma and in later stages. Furthermore, cognitive therapy theories based on the social cognitive theory have focused on the post-traumatic changes in individuals’ schemas [141,142]. As such, it is necessary to take a cognitive approach to understand the effects of a traumatic event on an individual and explore its meanings when attempting to provide counseling for PTSD. Teachers’ self-image and expectations of their job before the trauma should be examined, and interventions should be provided to address problems in these areas. Particularly, considering that Living a Calling alleviated the relationship between PTSD from violence victimization and the negative consequences, providing counseling with a focus on exploring the type of Calling teachers had prior to the trauma and enhancing their sense of Calling by reinforcing the meaning and value of their teaching career would curtail the adverse consequences of violence victimization among teachers. Cognitive behavioral therapy (CBT) [143], which deals with cognition and emotion to intervene intensively in memory and distorted cognitive schemas arising from trauma, would be an effective approach for this purpose. CBT theorizes that PTSD results from the individual’s interpretation of trauma instead of the trauma itself. Thus, analyzing teachers’ thinking processes, emotions, and behaviors and correcting them to more positive or reasonable directions may be effective in dealing with their trauma. In particular, dealing with the meaning of trauma and the underlying psychological meanings (such as safety, trust, power, self-esteem, and intimacy) with a focus on trauma-focused themes [144] to restore their pre-trauma schemas, as well as their positive self-image and Calling as teachers, would be an effective approach.

Third, this study found that social interaction anxiety and psychological burnout are important mediators; hence, counseling strategies must include effective approaches that deal with anxiety and burnout. Teachers who suffered violence may be anxious about being negatively evaluated by others despite being the victim. Moreover, teachers tend to have trouble seeking help and try to endure challenges due to their social position and expectations as teachers [145]. This may ultimately provoke anxiety in interpersonal relationships and psychological burnout. Thus, CBT that identifies the degree of social interaction anxiety and corrects irrational thinking that triggers such anxiety could be effective. In addition, self-care by teachers is important in preventing burnout. Calhoun and Tedeschi [146] recommended using mindfulness, meditation, and systematic relaxation techniques as some self-care strategies for counselors. These methods are useful for preventing burnout among teachers and may help relieve teachers’ stress and unpleasant feelings from violence victimization, thus preventing psychological burnout. Thus, incorporating mindfulness training in counseling for teachers who suffered violence could be helpful to effectively alleviate the adverse outcomes of PTSD.

Finally, this study provides several implications for various educational settings. First, the Act on the Prevention of and Countermeasures against Violence in Schools defines school violence as violence inflicted on a student [42]. That is, teachers are not protected against violence under this law. Several countries worldwide have implemented legal measures to protect teachers from violence. In the United States, students who defy a teacher’s instructions are subject to counseling with the dean, in addition to a mandatory meeting with their parents. Noncompliance results in punishment by a fine, and a violation of school regulations leads to punishment by a temporary or indefinite suspension. Students who threaten or physically assault a teacher are expelled from school and this is punishable by jail time [147]. Considering the damages and consequent repercussions of the infringement of teachers’ rights in Korea, the Korean government must also implement legal protection against violence against teachers, such that they can teach in a safe environment.

The limitations of this study and recommendations for future studies are as follows. First, this study viewed violence victimization among current teachers as trauma, but we could not include the mechanisms through which teachers overcome this trauma. Some individuals who experience trauma overcome it and achieve post-traumatic growth [148], and subsequent studies should explore the mechanisms through which current teachers overcome their trauma from violence victimization. Second, we included data from elementary, middle, and high school teachers, but the types of violence and the consequent adverse outcomes may differ across schools due to differences in students’ ages and school atmosphere. Examining elementary, middle, and high schools separately would yield more useful implications for each school level. Third, this study has a cross-sectional design; thus, the directionality of the variables cannot be determined. In particular, traumatic experiences may have long-term consequences; therefore, longitudinal studies with longer follow-up research are needed. Fourth, this study analyzed the effect by setting Living a Calling as the moderating variable; however, further research is needed to verify Living a Calling’s effect on workplace motivation. In addition, to retain teachers and help them manage their PTSD symptoms in a school environment, each type of trauma experience must be analyzed. Finally, this study is not free of the risk of common method bias. Subsequent studies should consider using different sources of data for the study variables to improve the objectivity of findings.

## Figures and Tables

**Table 1:** Means and standard deviations, correlations, and discriminant validity of the measurement variables.

Variable	1	2	3	4	5	6	7	8	M	SD	Skewness	Kurtosis
1. Violencevictimization	**(0.74)**								1.47	0.50	2.14	5.97
2. PTSD	0.56 **	**(0.85)**							2.29	1.04	0.24	−1.14
3. Socialinteractionanxiety	0.34 **	0.47 **	**(0.84)**						2.45	0.75	0.31	−0.30
4. Psychologicalburnout	0.38 **	0.42 **	0.51 **	**(0.67)**					3.06	0.96	0.08	−0.69
5. Turnoverintention	0.32 **	0.26 **	0.25 **	0.41 **	**(0.79)**				1.93	1.08	0.95	−0.19
6. Binge-eating	0.31 **	0.30 **	0.36 **	0.43 **	0.25 **	**(0.65)**			2.19	1.02	0.90	0.27
7. Problemdrinking	0.25 **	0.15 **	0.24 **	0.33 **	0.25 **	0.32 **	**(0.76)**		1.33	0.62	2.50	6.29
8. LivingaCalling	−0.17 **	−0.13 **	−0.21 **	−0.56 **	−0.38 **	−0.23 **	−0.11 *	(0.85)	4.81	1.37	−0.73	0.32

Note. *n* = 420. * p < 0.05. ** p < 0.01. The bold values in parentheses are the average variance extracted (AVE) values. Below the diagonal are the correlations among the constructs.

**Table 2:** Evaluation of the indirect effects.

Independent Variable → Mediator → Dependent Variable	Path Coefficient	95% CI (Min, Max)	
Violence victimization → PTSD → social interaction anxiety	0.379 **	0.318	0.481
Violence victimization → PTSD → psychological burnout	0.275 **	0.180	0.384
Violence victimization → PTSD → social interaction anxiety → psychological burnout	0.200 **	0.154	0.267
Violence victimization → PTSD → social interaction anxiety → binge-eating	0.052	0.001	0.116
Violence victimization → PTSD → social interaction anxiety → turnover intention	−0.004	−0.053	0.042
Violence victimization → PTSD → social interaction anxiety → problem drinking	0.020	−0.016	0.054
Violence victimization → PTSD → psychological burnout → binge-eating	0.112 **	0.068	0.185
Violence victimization → PTSD → psychological burnout → turnover intention	0.109 **	0.068	0.189
Violence victimization → PTSD → psychological burnout → problem drinking	0.066 *	0.038	0.111
Violence victimization → PTSD → social interaction anxiety → psychological burnout→ binge-eating	0.082 *	0.054	0.129
Violence victimization → PTSD → social interaction anxiety → psychological burnout→ turnover intention	0.079 *	0.053	0.124
Violence victimization → PTSD → social interaction anxiety → psychological burnout→ problem drinking	0.048 *	0.031	0.076

Note. *n* = 420. * p < 0.05. ** p < 0.01.

**Table 3:** Moderated mediating effect.

Conditional Indirect Effects	Independent Variable → Mediator → Dependent Variable	Index	BootSE	95% CI (Min, Max)	
Living a Calling	Violence victimization → PTSD → turnover intention	−0.1603 **	0.0478	−0.2372	−0.0821
	Violence victimization → PTSD → social interaction anxiety → turnover intention	0.0549 *	0.0200	0.0217	0.0878
	Violence victimization → PTSD → psychological burnout → turnover intention	−0.0024	0.0064	−0.0144	0.0064
	Violence victimization → PTSD → social interaction anxiety → psychological burnout → turnover intention	−0.0013	0.0035	−0.0076	0.0037
	Violence victimization → PTSD → binge-eating	0.0169	0.0592	−0.0788	0.1155
	Violence victimization → PTSD → social interaction anxiety → binge-eating	0.0166	0.0189	−0.0127	0.0487
	Violence victimization → PTSD → psychological burnout → binge-eating	0.0046	0.0074	−0.0059	0.0183
	Violence victimization → PTSD → social interaction anxiety → psychological burnout → binge-eating	0.0025	0.0040	−0.0030	0.0100
	Violence victimization → PTSD → problem drinking	0.0183	0.0393	−0.0440	0.0842
	Violence victimization → PTSD → social interaction anxiety → problem drinking	0.0186	0.0130	−0.0032	0.0394
	Violence victimization → PTSD → psychological burnout → problem drinking	0.0025	0.0044	−0.0037	0.0106
	Violence victimization → PTSD → social interaction anxiety → psychological burnout → problem drinking	0.0013	0.0025	−0.0018	0.0060

Note. *N* = 420. * p < 0.05. ** p < 0.01.

**Table 4:** Indirect effects of PTSD and social interaction anxiety on turnover intention according to Living a Calling.

Violence Victimization → PTSD → Social Interaction Anxiety → Turnover Intention					
Moderator	Living a Calling	Effect	BootSE	95% CI (Min, Max)	
M − 1SD	3.4413	−0.0570	0.0397	−0.1221	0.0082
M	4.8155	0.0184	0.0282	−0.0289	0.0651
M + 1SD	6.1897	0.0938	0.0391	0.0302	0.1584

Note. *n* = 420.

**Table 5:** Indirect effects of PTSD on turnover intention according to Living a Calling.

Violence Victimization → PTSD → Turnover Intention					
Moderator	Living a Calling	Effect	BootSE	95% CI (Min., Max.)	
M − 1SD	3.4413	0.2653	0.0940	0.1172	0.4245
M	4.8155	0.0451	0.0684	−0.0629	0.1586
M + 1SD	6.1897	−0.1751	0.0956	−0.3290	−0.0167

Note. *n* = 420.

## Data Availability

Data collected and analyzed during the study are available upon reasonable request.

## References

[1] Peist E., McMahon S.D., Davis J.O., Keys C.B. (2020). Teacher turnover in the context of teacher-directed violence: An empowerment lens. J. Sch. Violence.

[2] Korean Educational Development Institute (2021). Pre-Primary, Primary and Secondary Education Statistics: Retirement of Teachers. Ministry of Education: Seoul, Republic of Korea. https://www.oecd.org/education/school/2713221.pdf.

[3] KFTA Press Release (2021). Korean Federation of Teachers’ Association: Seoul, Republic of Korea. ttps://en.wikipedia.org/wiki/Korean_Federation_of_Teachers%27_Associations.

[4] Lee K.M., Shon K.S. (2013). A qualitative study about psychosocial sequela of teachers’ violence victimization. Korean J. Sch. Psychol..

[5] Lee K.M., Cho E.S. (2015). A study on the mediating factors on violence against teachers and their effects. Korean J. Counsel. Psychother..

[6] Wilson C.M., Douglas K.S., Lyon D.R. (2011). Violence against teachers: Prevalence and consequences. J. Interpers. Violence.

[7] Kim S.Y., Oh I.S. (2018). Influencing factors on post-traumatic growth among teachers who experience violence victimization as post-traumatic stress: Mediator of self-disclosure and rumination and moderator of social support. Teach Educ. Res..

[8] Cho Y.S. (2018). An analysis of differences in teacher’s psychological difficulty based on types of infringement of teacher right. J. Learn. -Cent. Curric. Instr..

[9] Holmes S.C., Johnson N.L., Johnson D.M. (2022). Understanding the relationship between interpersonal trauma and disordered eating: An extension of the model of psychological adaptation. Psychol. Trauma.

[10] Marshall-Berenz E.C., Vujanovic A.A., MacPherson L. (2011). Impulsivity and alcohol use coping motives in a trauma-exposed sample: The mediating role of distress tolerance. Pers. Indiv. Dif..

[11] Michael M.L., Witte T.H. (2021). Traumatic stress and alcohol-related disordered eating in a college sample. J. Am. Coll. Health.

[12] Weiss N.H., Tull M.T., Sullivan T.P., Dixon-Gordon K.L., Gratz K.L. (2015). Posttraumatic stress disorder symptoms and risky behaviors among trauma-exposed inpatients with substance dependence: The influence of negative and positive urgency. Drug Alcohol. Depend..

[13] Min B.H., Young C.J. (2021). A study on teachers’ psychological burnout research trends in Korea: Focusing on 2013–2020. CNU J. Educ. Stud..

[14] Calhoun L.G., Cann A., Tedeschi R.G., Weiss T., Berger R. (2010). The posttraumatic growth model: Sociocultural considerations. Posttraumatic Growth and Culturally Competent Practice: Lessons Learned from around the Globe.

[15] Karatuna I., Gök S. (2014). A study analyzing the association between post-traumatic embitterment disorder and workplace bullying. J. Workplace Behav. Health.

[16] Weiss H.M., Cropanzano R., Staw B.M., Cummings L.L. (1996). Affective events theory: A theoretical discussion of the structure, causes and consequences of affective experiences at work. Research in Organizational Behavior: An Annual Series of Analytical Essays and Critical Reviews.

[17] Contractor A.A., Weiss N.H. (2019). Typologies of PTSD clusters and reckless/self-destructive behaviors: A latent profile analysis. Psychiatry Res..

[18] Judge T.A., Locke E.A., Durham C.C., Kluger A.N. (1998). Dispositional effects on job and life satisfaction: The role of core evaluations. J. Appl. Psychol..

[19] Cho M.K., Shin H.C. (2021). The relationship among anxiety attachment and growth following romantic breakups of college students: The double mediation effects of intrusive rumination, deliberate rumination. Korean J. Couns. Psychother..

[20] Maslach C., Schaufeli W.B., Leiter M.P. (2001). Job burnout. Annu. Rev. Psychol..

[21] Kim Y.S., Lee J.S. (2017). The effects of trauma and intrusive rumination on posttraumatic stress disorder symptoms: The moderated moderated mediation of future prospective cognition and gender difference. Korean J. Clin. Psychol..

[22] Lim S.Y. (2014). The Effects of Negative Urgency, Negative Affect and Emotional Dysregulation on Binge Eating and Self Harm Behavior: In Terms of the UPPS-P Model. Ph.D. Thesis.

[23] Cha M.G., Kim E.K. (2020). The effect of post-traumatic stress disorder on firefighters’ turnover intention. Korean Assoc. Public Saf. Crim. Just Rev..

[24] Kim S.Y. (2018). The Relationship between Experience of Violence Victimization as Post-Traumatic Stress and Post-Traumatic Growth: Focusing on the Mediating Effect of Self-Disclosure and Rumination and the Moderation Effect of Social Support. Master’s Thesis.

[25] Klassen R.M., Tze V.M., Betts S.M., Gordon K.A. (2011). Teacher efficacy research 1998–2009: Signs of progress or unfulfilled promise?. Educ. Psychol. Rev..

[26] Liu S., Onwuegbuzie A.J. (2012). Chinese teachers’ work stress and their turnover intention. Int. J. Educ. Res..

[27] Briere J., Hodges M., Godbout N. (2010). Traumatic stress, affect dysregulation, and dysfunctional avoidance: A structural equation model. J. Trauma Stress.

[28] Contractor A.A., Caldas S., Weiss N.H., Armour C. (2018). Examination of the heterogeneity in PTSD and impulsivity facets: A latent profile analysis. Pers. Indiv. Dif..

[29] Yoo H.S., Hwang S.Y. (2020). A study on posttraumatic stress experience of special education teacher exposed to violence behaviors of students with disabilities. J. Emot. Behav. Disord..

[30] Lee J.Y. (2020). A Study on Post-Traumatic Stress and Post-Traumatic Growth of the Teachers in Counseling. Master’s Thesis.

[31] Ok J.H. (2009). A study of job satisfaction, organizational commitment and turnover intention influenced by job stress factors. J. Korean Teach Educ..

[32] Khu B.Y., Kim Y.M. (2014). A relationship among secondary school teachers’ stressors, psychological burnout, and teacher efficacy. Korean J. Youth Stud..

[33] Kim Y.J., Chung N.W. (2016). The mediating effect of acceptance on the relationship between emotional clarity and social interaction anxiety in college students. Korean J. Health Psychol..

[34] Lee Y.M. (2016). A meta-analysis on the variables related to teachers’ psychological burnout. Teach Educ. Res..

[35] Park J.E., Chung N.W. (2021). The influences of internalized shame on social interaction anxiety. J. Learn. Cent. Curric. Instr..

[36] Mattick R.P., Clarke J.C. (1998). Development and validation of measures of social phobia scrutiny fear and social interaction anxiety. Behav. Res. Ther..

[37] Yang C.H., Lee J.Y. (2020). A concept map of the difficulties and overcoming factors in response to the infringement of special school teachers’ rights. J. Learn. -Cent. Curric. Instr..

[38] Kuo J.R., Goldin P.R., Werner K., Heimberg R.G., Gross J.J. (2011). Childhood trauma and current psychological functioning in adults with social anxiety disorder. J. Anxiety Disord..

[39] Vassilopoulos S.P., Banerjee R. (2010). Social interaction anxiety and the discounting of positive interpersonal events. Behav. Cogn. Psychother..

[40] Safren S.A., Turk C.L., Heimberg R.G. (1998). Factor structure of the social interaction anxiety scale and the social phobia scale. Behav. Res. Ther..

[41] Lee E.H., Lee J.H. (2001). Relationship of teachers’ job stressors to psychological adjustment: The moderating effect of problem-focused coping and emotion-focused coping. Korean J. Health Psychol..

[42] Park K.A., Cho H.J. (2015). The study on infringement condition of teachers’ right and coping course of teachers’ bullying by victimization experience of school violence on teachers. Korean J. Teach. Educ..

[43] Bandalos D.L., Finney S.J., Marcoulides G.A., Schumacker R.E. (2001). Item parceling issues in structural equation modeling. New Developments and Techniques in Structural Equation Modeling.

[44] Jun I., Oh S.H., Sung H.Y. (2015). The effects of job insecurity among contract teachers on job stress and job attitude. J. Bus Res..

[45] Cho Y.K. (2019). A Qualitative Study on the Recovery Experience through Counseling and the Psychological Trauma of Teachers who Experienced Teachers’ Right Infringement. Ph.D. Thesis.

[46] Kwon O.H. (2010). The Relationship of Teachers’ Experiences with Violence by Students and Parents to Mental Health and Burnout. Master’s Thesis.

[47] Choi Y.R. (2016). The Relationship between Teachers’ Experiences of Violence against Teachers and Teacher Burnout: Verifying the Mediational Effect of Cognitive⋅Behavioral⋅Emotional Responses to Violence against Teachers. Master’s Thesis.

[48] Kim T.S., Lee J.Y. (2015). Understanding of teacher-targeted bullying and intervention plans. Korean J. Couns..

[49] Ford J.D., Grasso D.J., Elhai J.D., Courtois C.A. (2015). Posttraumatic Stress Disorder: Scientific and Professional Dimensions.

[50] Kang H.G. (1996). An Analysis of the Relationship between the Characteristics and Factors of Job Stress in Special Education School Teachers and General Education School Teachers. Ph.D. Thesis.

[51] Kim S.B. (2020). The Mediating Effects of Teacher Efficacy of the Relationship between Job-Stress and Burnout of Elementary School Teachers, who Provide Integrative Education to Children with ADHD. J. Spec. Educ. Rehabil..

[52] Yoo S.K., Park S.H. (2002). Influence of Occupational Stress and Perceived Social Support on Counselors’ Burnout in Korea. Korean J. Counsel. Psychother..

[53] Maslach C., Jackson S.E. (1981). The measurement of experienced burnout. J. Organ. Behav..

[54] Pietarinen J., Pyhältö K., Haverinen K., Leskinen E., Soini T. (2021). Is individual- and school-level teacher burnout reduced by proactive strategies?. Int. J. School Educ. Psychol..

[55] Shin H., Noh H., Jang Y., Park Y.M., Lee S.M. (2013). A longitudinal examination of the relationship between teacher burnout and depression. J. Employ Couns..

[56] Kang M.J., Namkoong S.E., Kim S.J., Koo M.H. (2003). Analysis of factors related in teacher’s problem drinking. J. Korean Acad. Psychiatr. Mental Health Nurs..

[57] Moenkemeyer G., Hoegl M., Weiss M. (2012). Innovator resilience potential: A process perspective of individual resilience as influenced by innovation project termination. Hum. Relat..

[58] Matsela M.A., Kirsten T. (2014). Teachers’ experiences and impact of workplace bullying on the health in Lesotho. Psychol. Res..

[59] Cho E.S. (2019). The Relationship between Positive Psychological Capital and Psychological Well-Being in Teachers with Experiences of Violence against Teachers: The Role of Self-Compassion and Social Support. Ph.D. Thesis.

[60] Berg J.M., Grant A.M., Johnson V. (2010). When Callings are Calling: Crafting Work and Leisure in Pursuit of Unanswered Occupational Callings. Organ Sci..

[61] Duffy R.D., Dik B.J. (2013). Research on calling: What have we learned and where are we going?. J Vocat Behav.

[62] Gazica M.W., Spector P.E. (2015). A comparison of individuals with unanswered callings to those with no calling at all. J. Vocat. Behav..

[63] Claes R., Quintanilla S.A.R. (1994). Initial career and work meanings in seven European countries. Career Dev. Q..

[64] Ahn J., Kim H.W., Lee J.Y. (2017). The structural relationship between the level of calling, job satisfaction and withdrawal intention: Focused on the mediating effects of living a calling, work meaning, and career commitment. J. Career Educ. Res..

[65] Lee E.K. (2011). A study on the perception of youth work as a vocation and attendant levels of job satisfaction. Stud. Korean Youth.

[66] Cardador M.T., Dane E., Pratt M.G. (2011). Linking calling orientations to organizational attachment via organizational instrumentality. J. Vocat. Behav..

[67] Jeong M.J., Kim J.W., Seo J.K. (2016). A narrative inquiry into teachers’ experience with restorative practices in schools. J. Anthropol. Educ..

[68] Dik B.J., Eldridge B.M., Steger M.F., Duffy R.D. (2012). Development and validation of the calling and vocation questionnaire (CVQ) and brief calling scale (BCS). J. Career Assess.

[69] Dobrow Riza S., Heller D. (2015). Follow your heart or your head? A longitudinal study of the facilitating role of calling and ability in the pursuit of a challenging career. J. Appl. Psychol..

[70] Jang J.Y., Lee J.Y. (2014). The relation between perceiving a calling and life satisfaction: The mediating effects of work meaning, living a calling, life meaning, and job satisfaction. Korean J. Counsel. Psychother..

[71] Duffy R.D., Bott E.M., Allan B.A., Torrey C.L., Dik B.J. (2012). Perceiving a calling, living a calling, and job satisfaction: Testing a moderated, multiple mediator model. J. Couns. Psychol..

[72] Ha Y.J. (2013). Calling and Work-Related Outcomes: Career Commitment as a Mediator and Person-Supervisor Fit and Perceived Organizational Support as Moderators. Ph.D. Thesis.

[73] Choi Y.R., Seo Y.S. (2022). The relationship between violence against teachers and burnout. J. Learn. Cent. Curric. Instr..

[74] Lim J.I., Sohn Y.W. (2016). The influence of the military junior leader’s relationship conflict on turnover intention—The moderated mediating effect of defensive silence and calling. J. Hum Resour. Manag. Res..

[75] Jeong H.K., Lee J.Y. (2020). Structural relationship analysis of teacher’s calling, P-E fit, career commitment, living calling, burnout and job satisfaction: Focus on the WCT Model. Asian J. Educ..

[76] Hwang E.C., Kim J.H., You J.Y., Song N.O., Shin H.J. (2020). Interpersonal problem and teacher burnout among teachers who experience violence victimization: The mediating effects of self-compassion. J. Learn. Cent. Curric. Instr..

[77] Choi H.Y. (2020). Moderating effect of vocational calling on firefighters’ stress and burnout. Fire Sci. Eng..

[78] Bakker A.B., Van Emmerik H., Van Riet P. (2008). How job demands, resources, and burnout predict objective performance: A constructive replication. Anxiety Stress Coping.

[79] Duffy R.D., Allan B.A., Bott E.M. (2012). Calling and life satisfaction among undergraduate students: Investigating mediators and moderators. J. Happiness Stud..

[80] Demerouti E., Bakker A.B., Leiter M. (2014). Burnout and job performance: The moderating role of selection, optimization, and compensation strategies. J. Occup. Health Psychol..

[81] Duffy R.D., Dik B.J., Douglass R.P., England J.W., Velez B.L. (2018). Work as a calling: A theoretical model. J. Couns. Psychol..

[82] Han B.C. (2012). Fatigue Society.

[83] Seol K.O., Im J.I. (2013). Collective self-esteem, calling and burnout among youth companions. Korean J. Counsel. Psychother..

[84] Jeong Y.H., Yu H.K. (2015). A study on direction of prevention and intervention for psychological healing of teachers experiencing infringement of teacher right. J. Learn. -Cent. Curric. Instr..

[85] Kim G.Y. (2021). The Effect of Elementary School Teachers’ Experiences with Violence by Students and Parents on Psychological Burnout: Moderating Effect of Self-Soothing Abilities. Master’s Thesis.

[86] Hayes A.F. (2018). Introduction to Mediation, Moderation, and Conditional Process Analysis: A Regression-Based Approach.

[87] De Wet C. (2010). Victims of educator-targeted bullying: A qualitative study. S. Afr. J. Educ..

[88] Faul F., Erdfelder E., Lang A.-G., Buchner A. (2007). G*Power 3: A flexible statistical power analysis program for the social, behavioral, and biomedical sciences. Behav. Res. Methods.

[89] Lee S.K. (2019). The actual condition of violence victimization experience of teachers and relationship with mental health, psychological well-being. J. Learn. -Cent. Curric. Instr..

[90] Eun H.J., Kwon T.W., Lee S.M., Kim T.H., Choi M.R., Cho S.J. (2005). A study on reliability and validity of the Korean version of Impact of Event Scale-Revised. J. Korean Neuropsychiatr. Assoc..

[91] Horowitz M., Wilner N., Alvarez W. (1979). Impact of Event Scale: A measure of subjective stress. Psychosom. Med..

[92] Weiss D.S., Marmar C.R., Wilson J.P., Keane T.M. (1997). The impact of event scale-revised. Assessing Psychological Trauma and PTSD: A Handbook for Practitioners.

[93] Kim B.R., Lee D.H., Lee D.Y., Lee D.H. (2019). The relationship of trauma event experience on psychological and PTSD symptoms, and posttraumatic growth: Mediating effect of stress coping ability. Korean J. Health Psychol..

[94] Lee S.Y., Lee D.H., Lee D.H., Lee M.Y. (2019). The structural relationship among optimism, coping, posttraumatic growth, PTSD symptoms, and drinking problems in adults who have experienced trauma. Korean J. Counsel. Psychother..

[95] Kim H.S. (2001). Memory Bias in Subtypes of Social Phobia. Master’s Thesis.

[96] Garner D.M., Olmsted M.P. (1984). Manual for Eating Disorder Inventory (EDI).

[97] Garner D.M. (1991). Eating Disorder Inventory—2: Professional Manual.

[98] Cho S.H. (2004). The Effects of Emotional Intensity and Emotional Clarity on Self-Destructive Behaviors: Focused on Binge Eating and Addictive Internet Use. Master’s Thesis.

[99] Lee I.S. (1998). The Effect of Restrained Eating on Eating Behavior. Ph.D. Thesis.

[100] Heo E.J. (2015). The Effects of Emotional Clarity on Self-Destructive Behaviors: Mediating Effect of Impulsivity. Master’s Thesis.

[101] Allen J.P., Reinert D.F., Volk R.J. (2001). The alcohol use disorders identification test: An aid to recognition of alcohol problems in primary care patients. Prevene Med..

[102] Lee B.O., Lee C.H., Lee P.G., Choi M.J., Namkoong K. (2000). Development of Korean version of Alcohol Use Disorders Identification Test (AUDIT-K): Its reliability and validity. J. Korean Acad. Addict. Psychiatry.

[103] Landau J., Hammer T.H. (1986). Clerical employees’ perceptions of intraorganizational career opportunities. Acad. Manag. J..

[104] Lee S.H., Kim M.S., Park S.K. (2008). A test of work-to-family conflict mediation hypothesis for effects of family friendly management on organizational commitment and turnover intention. Korean J. Ind. Organ. Psychol..

[105] Seo Y.S. (2010). Testing mediator and moderator effects in counseling psychology research: Conceptual distinction and statistical considerations. Korean J. Counsel. Psychother..

[106] MacCallum R.C., Widaman K.F., Zhang S., Hong S. (1999). Sample size in factor analysis. Psychol. Method.

[107] Bandalos D.L. (2008). Is parceling really necessary? A comparison of results from item parceling and categorical variable methodology. Struct. Equ. Model..

[108] Fornell C., Larcker D.F. (1981). Evaluating structural equation models with unobservable variables and measurement error. J. Market Res..

[109] Hult G.T.M., Hair J.F., Proksch D., Sarstedt M., Pinkwart A., Ringle C.M. (2018). Addressing endogeneity in international marketing applications of partial least squares structural equation modeling. J. Int. Market.

[110] Darlington R.B., Hayes A.F. (2001). Regression Analysis and Linear Models: Concepts, Applications, and Implementation.

[111] Hayes A.F., Montoya A.K., Rockwood N.J. (2017). The analysis of mechanisms and their contingencies: PROCESS versus structural equation modeling. Australas. Mark J..

[112] West S., Finch J., Curran P., Hoyle R. (1995). Structural equation models with non-normal variables: Problems and remedies. Structural Equation Modeling: Concepts, Issues, and Applications.

[113] Finch F.F., West S.G., MacKinnon D.P. (1997). Effects of Sample Size and Non-Normality on the estimation of mediated effects in latent variable models. Struct. Equ. Model..

[114] Podsakoff P.M., Organ D.W. (1986). Self-reports in organizational research: Problems and prospects. J. Manag..

[115] Yu J.P. (2015). The Concept and Understanding of Structural Equation Modeling.

[116] Martens M. (2005). The use of structural equation modeling in counseling psychology research. Couns. Psychol..

[117] West S.G., Aiken L.S., Krull J.L. (1996). Experimental personality designs: Analyzing categorial by continuous variable interactions. J. Pers..

[118] Yoo H.S., Kwon J.H. (2017). Structural relations among child care teachers’ job stress, teacher efficacy, organizational commitment, burn-out and turnover intention. Early Child Educ. Res. Rev..

[119] Cho S.M., Chung H.S., Han Y.S. (2020). The effects of mental violence on turnover intention in social workers: Focused on the mediating effects of job stress and burnout. Health Soc. Welf. Rev..

[120] Medisauskaite A., Kamau C. (2019). Does occupational distress raise the risk of alcohol use, binge-eating, ill health and sleep problems among medical doctors? A UK cross-sectional study. BMJ Open..

[121] Duan X., Ni X., Shi L., Zhang L., Ye Y., Mu H., Li Z., Liu X., Fan L. (2019). The impact of workplace violence on job satisfaction, job burnout, and turnover intention: The mediating role of social support. Health Qual. Life Outcomes.

[122] Liu W., Zhao S., Shi L., Zhang Z., Liu X., Li L., Duan X., Li G., Lou F., Jia X. (2018). Workplace violence, job satisfaction, burnout, perceived organisational support and their effects on turnover intention among Chinese nurses in tertiary hospitals: A cross-sectional study. BMJ Open..

[123] Yang Y.H., Kim J.K. (2016). Factors influencing turnover intention in clinical nurses: Compassion fatigue, coping, social support, and job satisfaction. J. Korean Acad. Nurs. Admin.

[124] Zhao S.H., Shi Y.U., Sun Z.N., Xie F.Z., Wang J.H., Zhang S.E., Gou T.Y., Han X.Y., Sun T., Fan L.H. (2018). Impact of workplace violence against nurses’ thriving at work, job satisfaction, and turnover intention: A cross-sectional study. J. Clin. Nurs..

[125] Kachadourian L.K., Pilver C.E., Potenza M.N. (2014). Trauma, PTSD, and binge and hazardous drinking among women and men: Findings from a national study. J. Psychiatr. Res..

[126] Paltell K.C., Smith R.L., Kansky J., Cox C.M., Amstadter A.B., Dick D., Salvatore J.E., Berenz E.C., Spit for Science Working Group (2020). Posttraumatic stress disorder symptoms, relationship quality, and risky alcohol use among trauma-exposed students. Addict. Behav..

[127] McGeary C.A., Garcia H.A., McGeary D.D., Finley E.P., Peterson A.L. (2014). Burnout and coping: Veterans Health Administration posttraumatic stress disorder mental health providers. Psychol. Trauma.

[128] Rosenbaum D.L., White K.S. (2013). The role of anxiety in binge-eating behavior: A critical examination of theory and empirical literature. Health Psychol. Res..

[129] Bothma C.F., Roodt G. (2013). The validation of the turnover intention scale. SA J. Hum. Resour. Manag..

[130] Cohen G., Blake R.S., Goodman D. (2016). Does turnover intention matter? Evaluating the usefulness of turnover intention rate as a predictor of actual turnover rate. Rev. Public Pers. Adm..

[131] Esteves T., Lopes M.P. (2017). Crafting a calling: The mediating role of calling between challenging job demands and turnover intention. J. Career Dev..

[132] Yang H.J., Han S.J. (2018). The calling, emotional exhaustion and turnover intention in hospital nurses. J. Humanit. Soc. Sci..

[133] Elangovan A.R., Pinder C.C., McLean M. (2010). Callings and organizational behavior. J. Vocat. Behav..

[134] Kahn R.L., Wolfe D.M., Quinn R.P., Snoek J.D., Rosenthal R.A. (1964). Organizational Stress: Studies in Role Conflict and Ambiguity.

[135] Duffy R.D., Sedlacek W.E. (2007). The presence of and search for a calling: Connections to career development. J. Vocat. Behav..

[136] Galand B., Lecocq C., Philippot P. (2007). School violence and teacher professional disengagement. Br. J. Educ. Psychol..

[137] Dzuka J., Dalbert C. (2007). Student violence against teachers: Teachers’ well-being and the belief in a just world. Eur. Psychol..

[138] Newman K., Fox C., Roth W., Mehta J., Harding D. (2004). Rampage: The Social Roots of School Shooters.

[139] Shin J.E., Oh I.S. (2017). Moderating effects of ego-resiliency and coping on violence against teachers and their mental health problems. J. Educ. Stud..

[140] Ehlers A., Clark D.M. (2000). A cognitive model of posttraumatic stress disorder. Behav. Res. Ther..

[141] Janoff-Bulman R. (1992). Shattered Assumptions: Towards a New Psychology of Trauma.

[142] McCann I.L., Pearlman L.A. (1990). Vicarious traumatization: A framework for understanding the psychological effects of working with victims. J. Trauma Stress.

[143] Beck A.T., Emery G., Greenberg R.L. (1985). Anxiety Disorders and Phobias: A Cognitive Perspective.

[144] Shipherd J.C., Street A.E., Resick P.A., Follette V.M., Ruzek J.I. (2006). Cognitive therapy for posttraumatic stress disorder. Cognitive-Behavioral Therapies for Trauma.

[145] Lee H. (2008). An Analysis of the Relationship of Social Support to Job Stress and Burnout in Elementary School Teachers. Master’s Thesis.

[146] Calhoun L.G., Tedeschi R.G. (2012). Posttraumatic Growth in Clinical Practice.

[147] Lee J.S., Cho M.H. (2017). A study on legal measures concerning violence against teachers by students and parents, etc. J. Hum. Rights Law Educ..

[148] Tedeschi R.G., Park C.L., Calhoun L.G. (1998). Posttraumatic Growth: Positive Changes in the Aftermath of Crisis.

